# Symmetrical D–π–A–π–D indanone dyes: a new design for nonlinear optics and cyanide detection

**DOI:** 10.3762/bjoc.22.6

**Published:** 2026-01-14

**Authors:** Ergin Keleş, Alberto Barsella, Nurgül Seferoğlu, Zeynel Seferoğlu, Burcu Aydıner

**Affiliations:** 1 Department of Chemistry, Graduate School of Natural and Applied Sciences, Gazi University, Yenimahalle, Ankara 06560, Türkiye; 2 Department of Chemistry, Faculty of Science, Gazi University, Yenimahalle, Ankara 06560, Türkiye; 3 Département d'Optique ultrarapide et Nanophotonique, IPCMS, UMR CNRS 7504, Université de Strasbourg, 23 rue du Loess, BP 43, 67034 Strasbourg Cedex 2, France; 4 Department of Advanced Technology, Graduate School of Natural and Applied Sciences, Gazi University, Yenimahalle, Ankara 06560, Türkiye

**Keywords:** chemosensor, DFT calculations, donor–π–acceptor–π–donor based organic dyes, indan-2-one, NLO

## Abstract

Three indan-2-one-based donor–π–acceptor–π–donor type dyes with symmetric donor groups were synthesized and characterized to study their nonlinear optical (NLO) properties and their potential use in the rapid and selective determination of cyanide. The designed structures feature symmetrical alkylaminophenyl donor groups and a strong electron-withdrawing dicyanovinylene as an acceptor connected through vinyl groups as a π-bridge. These strongly π-conjugated organic dyes can absorb in the NIR region, and they showed sensitivity towards the polarity of solvents with colorimetric and optical changes. Because of the strong donor–acceptor structure, second-order NLO properties were studied by measuring electric field-induced second harmonic (EFISH) values, which showed significant second-order NLO responses. The experimental results were explained using density functional theory (DFT) methods. The dyes also exhibit chemosensor properties, showing selectivity for cyanide via a Michael addition mechanism that causes the disappearance of the ICT band, and a significant color change was observed in both organic and aqueous media. In addition, the interaction mechanism between cyanide and the chemosensor is determined by a ^1^H NMR study and explained by DFT calculations.

## Introduction

Over the past decades, the functional heterocyclic push–pull dyes have attracted significant attention due to their widespread use in materials chemistry. This type of dye is of particular interest in the fields of organic electronics, photonics, and optoelectronics etc., used in areas such as dye-sensitized solar cells (DSSC), organic light-emitting diodes (OLED), nonlinear optics (NLO), and organic semiconductors and are also broadly used in diagnostic kits for diseases, fluorescence sensors, and various biotechnological fields [1–7]. Especially, organic materials showing nonlinear optical (NLO) properties have considerable advantages, such as low-cost production and larger NLO responses over inorganic counterparts [8–10]. Conjugated organic molecules containing electron-donating and accepting groups exhibit higher second-order nonlinearity due to having planar structures, long π-conjugations, and thermal stability [11]. Organic dyes that display efficient second-order NLO have high hyperpolarizability (β) values through electron-donor (D) and -withdrawing (A) groups linked by π-bridges in their structures [12–13]. Thus, NLO responses can be tunable by adjusting the strength of the donor and acceptor groups based on the intramolecular charge transfer (ICT) efficiency [14–16]. Indanones are highly conjugated with a planar structure, which favors overlap between the molecules. They are building blocks for many compounds, such as organic materials for optoelectronic and NLO applications [17–18]. Research shows that the absorption wavelength of the region can be shifted in the deep red/NIR region by changing the donor group with increased conjugated systems [19–22]. Gupta et al. studied optical properties of D–π–A system-based indan-2-one derivatives, and symmetric derivatives showed a significant bathochromic shift (≈300 nm) compared to asymmetric ones [23].

Organic dyes are also used as chemosensors, which provide economical, fast, and equipment-free analysis for the detection of environmental pollutants affecting the environment and human health [24–25]. Colorimetric detection of specific ions like cyanide, which is considered a highly toxic anion and dangerous to human health, by dyes gives advantages such as high sensitivity, fast response, low cost, and ease of operation [26–28].

**Figure 1:**
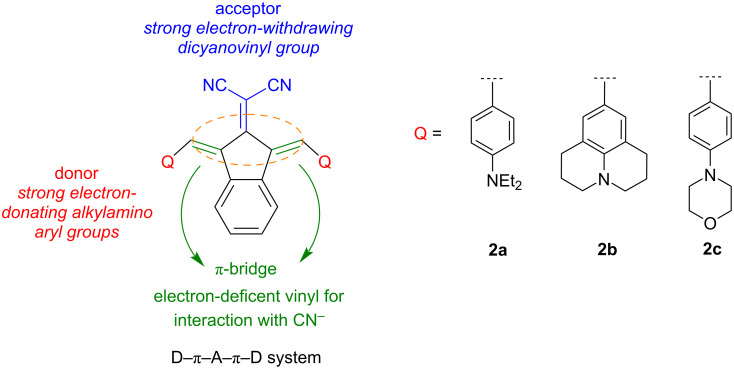
Design of the functional dyes.

In this study, symmetric novel indan-2-one derivatives with a D–π–A–π–D system were synthesized, and their structures were characterized using ^1^H NMR, ^13^C NMR, and mass spectrometry methods. Dye structures were designed with alkylaminophenyl groups, known for their various electron-donating properties, as donors, and the dicyanovinyl group, with its strong electron-withdrawing properties, as acceptors, conjugated with an indan-2-one core (Figure 1). The vinyl bridges were added to the design, which have the potential to open nucleophilic addition reactions (Michael type) due to their electron deficiencies, in addition to acting as π-bridges for the D–A conjugation. Furthermore, a symmetric design with increased π-conjugation was planned to shift the absorption bands towards the NIR region. Syntheses of dyes were carried out using the conventional method and the microwave irradiation (MWI) method, and the results were compared with yields and reaction times. Absorption spectra in solvents of different polarities were examined to determine the photophysical properties of the dyes. The sensitivity/selectivity properties of the dyes to anions were investigated in DMSO and aqueous media, revealing that the dyes were selectively responsive only to cyanide anions. Changes after interactions were determined through absorption spectra and color changes under ambient light. In addition, interaction mechanisms of dyes with cyanide were studied using the ^1^H NMR titration method, and it was determined that they interacted through an addition mechanism. Photophysical properties and interaction mechanisms of the compounds were also supported through density functional theory (DFT) and time-dependent DFT (TD-DFT) calculations, which were consistent with experimental results. NLO properties of the compounds were experimentally determined using the EFISH method and calculated using theoretical methods. Additionally, the thermal decomposition temperatures, an important parameter for compounds used in electro-optic (EO) application, were determined by thermogravimetric analysis (TGA).

## Results and Discussion

### Syntheses

**Scheme 1:**
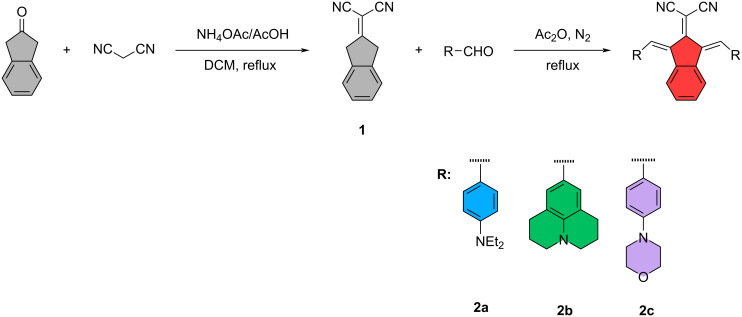
Synthetic pathway of compounds.

In the first step, 2-(1,3-dihydro-2*H*-inden-2-ylidene)malononitrile (**1**) was synthesized by indan-2-one and malononitrile in DCM with ammonium acetate/acetic acid buffers with a good yield (84%). Target compounds were synthesized by a coupling reaction between 2-(1,3-dihydro-2*H*-inden-2-ylidene)malononitrile (**1**) and appropriate alkylaminobenzaldehyde derivatives in acetic anhydride. Compounds were obtained with low to good yields (25–75%, conventional method (CM)) (Scheme 1, Table 1). All syntheses were also carried out with microwave irradiation (MWI), which had a short reaction time; however, no improvement in yield was achieved (see Supporting Information File 1). The structural analyses of the compounds were performed using ^1^H/^13^C NMR and mass spectrometry methods (Figures S1–S10 in Supporting Information File 1).

**Table 1:** Summary of reaction conditions, yields, and solid state color of dyes **2a–c**.

Dyes	CM	MWI	Color

	Yield (Time)	Yield (Time)	

**2a**	75% (45 min)	62% (4.5 min)	dark purple
**2b**	46% (30 min)	45% (4.5 min)	dark green
**2c**	22% (2 h)	25% (4.5 min)	black

### Optical properties of dyes

**Figure 2:**
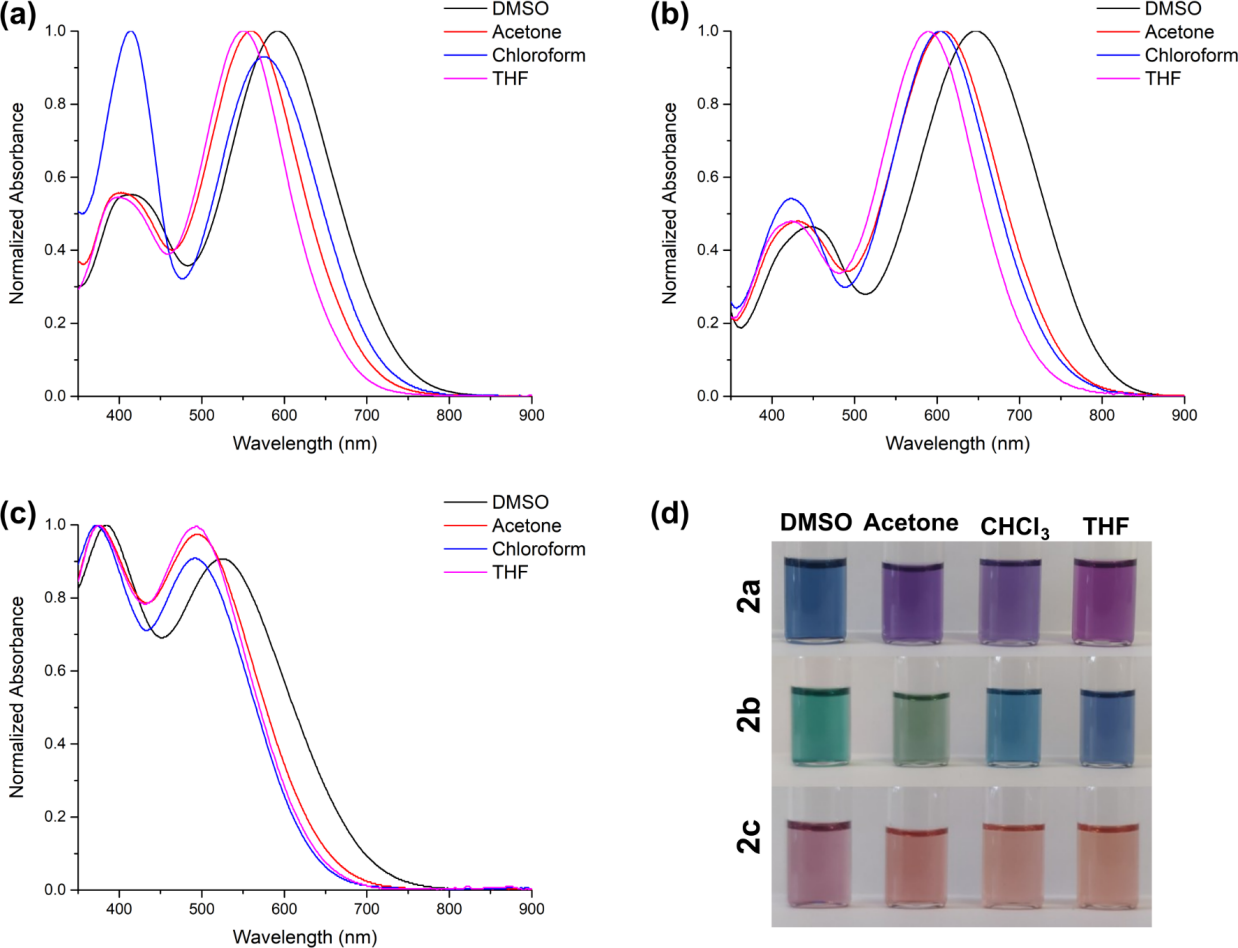
Normalized absorption spectra of dyes **2a** (a), **2b** (b), and **2c** (c); Photographs of dyes in the given solvents of different polarities under ambient light (d) *c* = 10 μM.

Photophysical properties of dyes **2a–c** were assessed in four different organic solvents with various polarities (DMSO, acetone, chloroform, and THF) via absorption spectra and DFT calculations (Table 2). Figure 2a–c displays the absorption spectra of the dyes and photographs under daylight in organic solvents (Figure S11 in Supporting Information File 1). The dyes exhibited two distinct absorption maxima in the range of 358–446 nm and 493–648 nm. The shorter wavelength absorption maxima are assigned the n–π* transition from the donor group to the dicyanovinyl, and longer wavelength absorption maxima are the n–π* transition of the conjugated structure [2].

**Table 2:** Photophysical properties of dyes **2a–c** in various solvents with different polarity and the calculated absorption spectra data^a^.

		Experimental		DFT calculations		

	Solvent	λ_Abs_ (nm)	ε (M^−1^⋅cm^−1^)	λ_Abs_ (nm)	*f*	Transitions, *w* (%)^b^

**2a**	DMSO	591	18413	607	0.8465	HOMO−1→LUMO, 99%
		410		422	0.7386	HOMO→LUMO+1, 99%

	acetone	560	16474	602	0.8231	HOMO−1→LUMO, 99%
		402		421	0.7418	HOMO→LUMO+1, 99%

	chloroform	583	14978	589	0.8191	HOMO−1→LUMO, 99%
		413		421	0.7639	HOMO→LUMO+1, 99%

	THF	550	19031	595	0.8209	HOMO−1→LUMO, 99%
		399		421	0.7540	HOMO→LUMO+1, 98%

**2b**	DMSO	648	19698	617	0.8785	HOMO−1→LUMO, 99%
		446		427	0.7331	HOMO→LUMO+1, 98%

	acetone	608	10470	612	0.8526	HOMO−1→LUMO, 99%
		430		426	0.7369	HOMO→LUMO+1, 98%

	chloroform	604	12566	598	0.8477	HOMO−1→LUMO, 99%
		423		425	0.7607	HOMO→LUMO+1, 98%

	THF	589	12650	604	0.8498	HOMO−1→LUMO, 99%
		424		426	0.7500	HOMO→LUMO+1, 99%

**2c**	DMSO	523	8103	572	0.5901	HOMO−1→LUMO, 3% HOMO→LUMO, 96%
		358		412	0.7351	HOMO−1→LUMO+1, 3% HOMO→LUMO+1, 96%

	acetone	494	7649	569	0.5829	HOMO−1→LUMO, 96% HOMO→LUMO, 3%
		377		411	0.5829	HOMO−1→LUMO+1, 3%
		491	8194	560	0.6331	HOMO→LUMO+1, 96%

	chloroform	491	8194	560	0.6331	HOMO−1→LUMO, 97% HOMO→LUMO, 2%
		372		411	0.7689	HOMO→LUMO+1, 97%

	THF	493	8784	564	0.6114	HOMO−1→LUMO, 97% HOMO→LUMO, 2%
		376		411	0.7561	HOMO−1→LUMO+1, 2% HOMO→LUMO+1, 97%

^a^ε was calculated according to the underlined band. ^b^HOMO: highest occupied molecular orbital, LUMO: lowest unoccupied molecular orbital.

Alkylamino groups (diethylamino, julolidine, and morpholine) as donor groups in chromophores have different electron-donor properties, causing a shift in the absorption wavelengths due to ICT transition to the dicyanovinylene acceptor [29–31]. The dyes show a bathochromic shift in absorption maxima, with the increased electron-donating tendency (Figure 2 and Table 2). Dyes **2a** and **2c** have free rotating alkylamino groups as diethylamino and morpholine, respectively, while **2b** have julolidine groups with restricted rotation, which makes the structure more planar. The absorption maxima are observed in order as **2b** > **2a** > **2c**. Therefore, these structural differences in donor groups showed that shifts towards longer wavelength absorption are attributed to the rigid planar structure, causing more delocalization of the π-electrons between donors and the acceptor (dicyanovinylene) group.

The effect of solvent media was investigated with solvents of varying polarities, which showed a bathochromic shift in the absorption maxima with increasing solvent polarity.

Dyes also showed significant color changes with increasing polarity. Color changes of **2a**; from purple to blue, **2b**; blue to green, and **2c**; pale orange to pale pink (Figure 2d). Dyes do not show any significant emission.

### Chemosensor properties

#### Cyanide selectivity study

**Figure 3:**
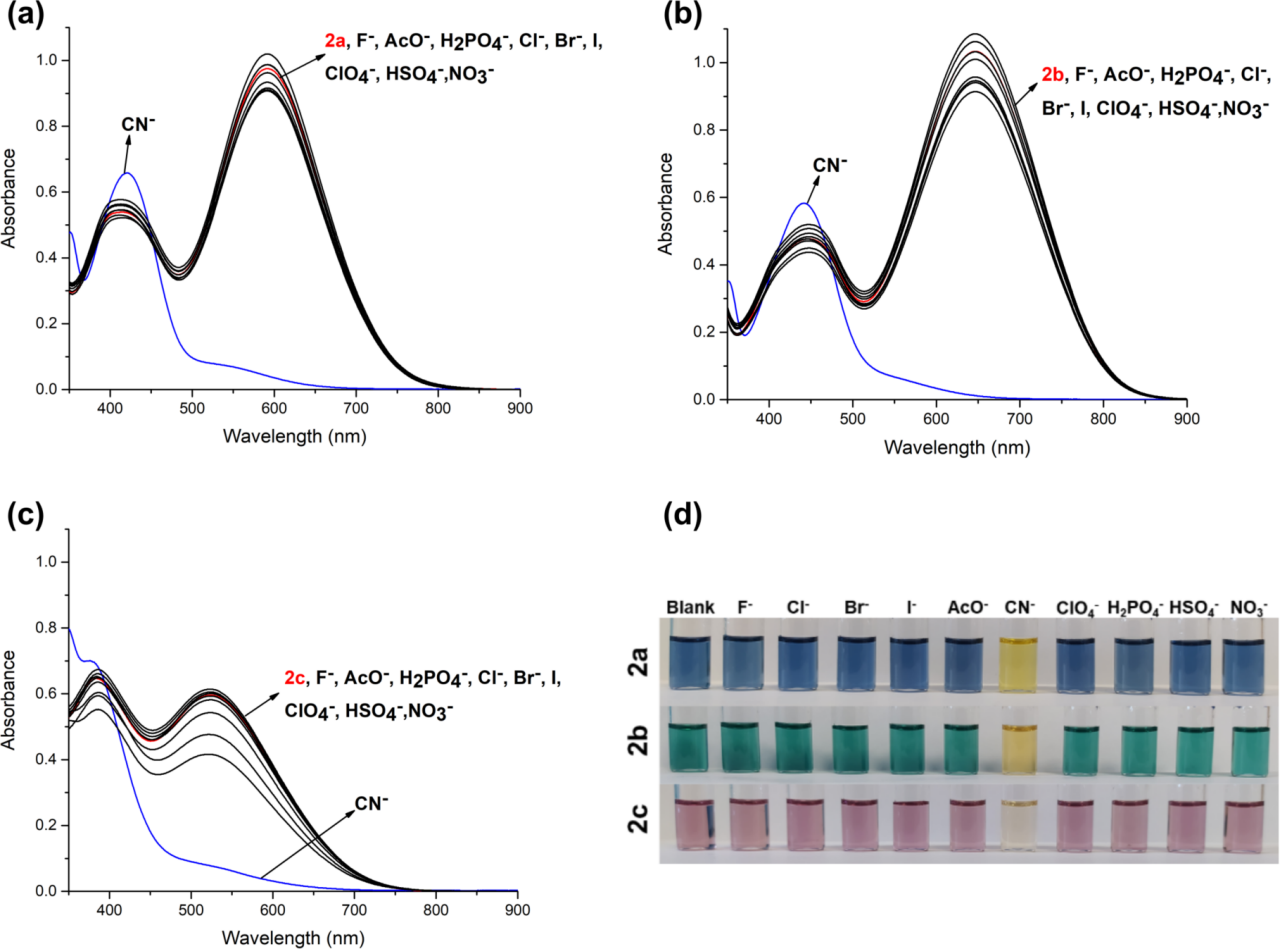
Absorption spectra of dyes **2a** (a), **2b** (b), and **2c** (c) upon addition of 20 equiv of anions in DMSO; photographs of dyes with/without addition of anions under ambient light (d) (*c* = 10 μM).

The dyes **2a**–**c** could have the ability to detect cyanide anions due to the presence of vinyl groups, where cyanide can be attacked via nucleophilic addition reaction. Therefore, the sensitivity and selectivity of dyes towards cyanide were investigated by the addition of cyanide (CN^−^) and competing anions (F^−^, Cl^−^, Br^−^, I^−^, AcO^−^, ClO_4_^−^, H_2_PO_4_^−^, HSO_4_^−^, and NO_3_^−^) in the form of the corresponding tetrabutylammonium (TBA) salt. Firstly, a titration study was conducted by the addition of 20 equiv of anions to dyes in organic solvent as DMSO (Figure 3). A significant response was only observed during the addition of cyanide. Upon addition of cyanide to dyes, the absorption bands at longer wavelength, 550–700 nm, disappeared while shorter wavelength absorption maxima, 350-450 nm, showed a slight increment in absorbance. The disappearance of the ICT band indicates that the conjugation through the structure of dyes between the donor and acceptor groups is disrupted. These results strongly suggest the addition of cyanide to the vinyl bridge. Furthermore, the color of dyes **2a**–**c**, blue, green, and pink, respectively, under ambient light changed to yellow when interacting with cyanide. The interaction mechanism was determined by ^1^H NMR, and the proposed mechanism was investigated in more detail by DFT studies. It is given in the next section.

**Figure 4:**
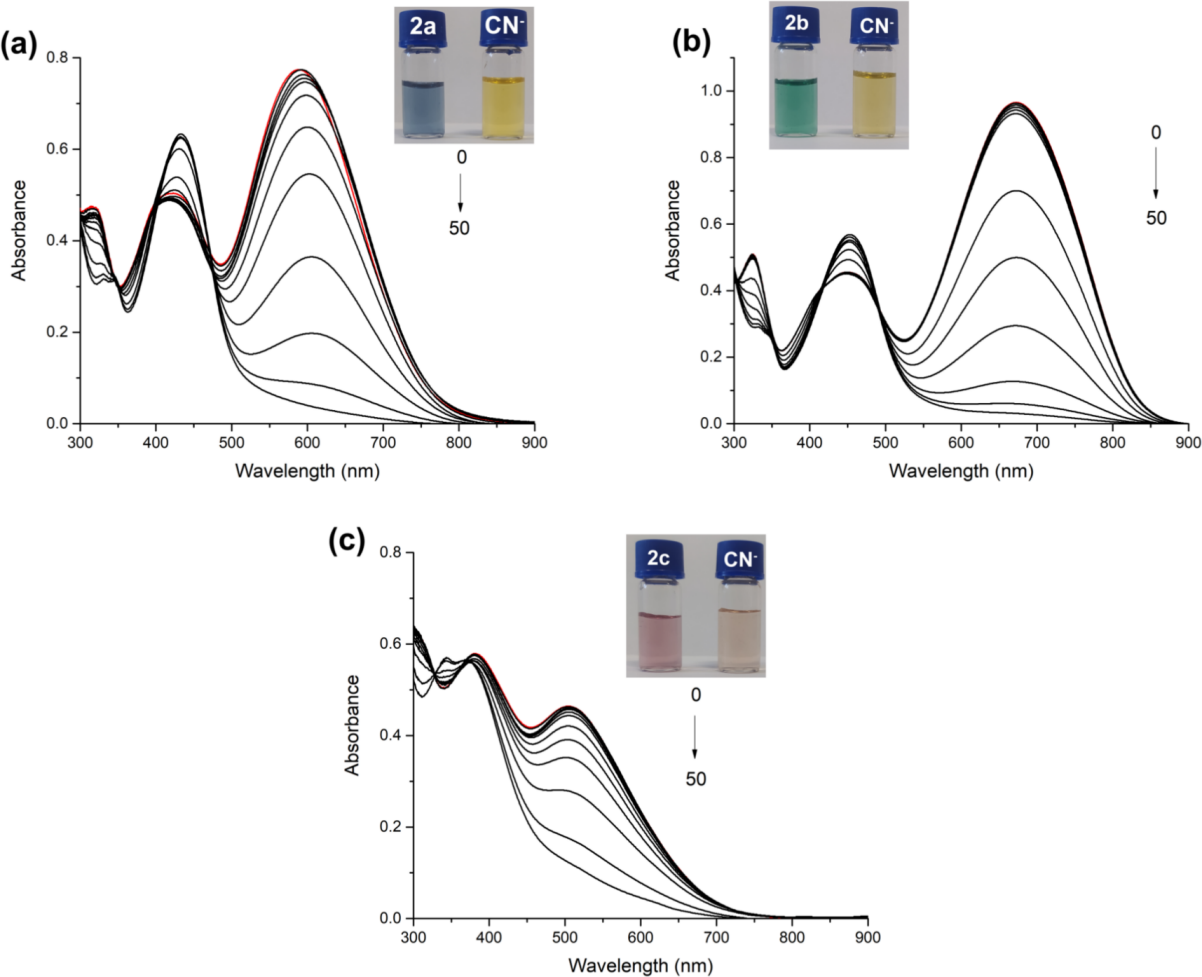
Absorption spectra of titrated dyes (**2a**–**c**) with up to 50 equiv of CN^−^ (a) 6:4, (b) 7:3, and (c) 4:6 in DMSO/H_2_O (v/v) (*c* = 30 μM).

In order to examine the sensitivity of the dyes to anions in aqueous media, firstly, the best ratio with DMSO as a co-solvent for aqueous medium was determined. For this purpose, absorption spectra were obtained by adding 50 equiv of CN^−^ to the DMSO/H_2_O (v/v) solution mixtures at certain ratios (*c* = 30 µM) (Figures S12–S14 in Supporting Information File 1). This study was applied separately for all dyes, and the appropriate ratio was determined for each as **2a**; 6:4, **2b**; 7:3, and **2c**; 4:6, DMSO/H_2_O, v/v. When the dyes were titrated with CN^−^ anions, the longer wavelength band decreased and disappeared, similar to the DMSO media (Figure 4). This result indicates that the interaction mechanism is similar in the aqueous environment. In addition, when the photographs taken with different solvents of the dyes were examined, similar changes in their colors were observed under daylight like those in DMSO (Figure 4, insets).

Additionally, the limit of detection (LOD) values of the compounds were calculated as 4.41 µM for **2a**, 9.49 µM for **2b** and 1.33 µM for **2c** in DMSO/H_2_O binary solvent medium (Figure S15 in Supporting Information File 1).

#### Interaction mechanism

**Figure 5:**
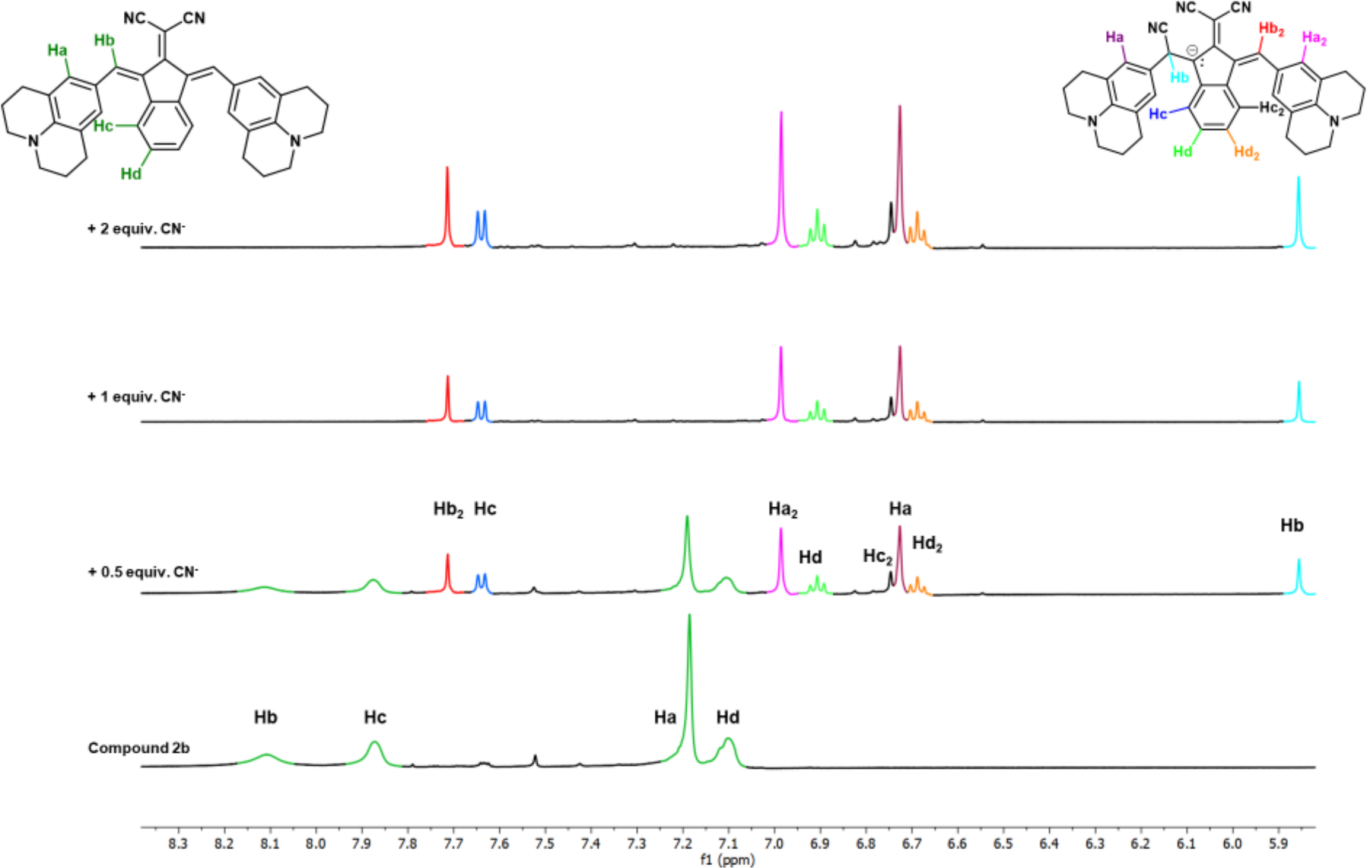
Partial ^1^H NMR spectral change of **2b** (*c* = 10 mM) after up to 2 equiv of TBACN (*c* = 1 M) in DMSO-*d*_6_.

**Scheme 2:**
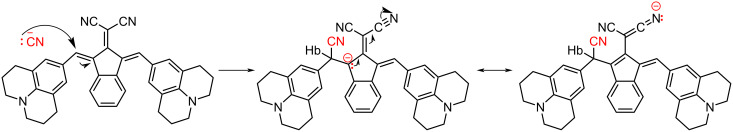
Proposed interaction mechanism with CN^−^.

^1^H NMR titration was performed to determine the interaction mechanism of the dyes (Figure 5). Upon the addition of 0.5 equiv of CN^−^, the intensity of the signals of **2b** decreased (green) while new signals appeared at the upfield, indicating the formation of another structure with increased electron density in conjugated system. Especially, the signal at 5.9 ppm (Hb, green to cyan) can be attributed to a change in aromatic hydrogen to aliphatic. The addition of cyanide to vinyl carbon can explain this change. Upon the addition of 1 equiv of CN^−^, signals of **2b** protons have completely disappeared, and the protons of the new species become apparent. An increase in cyanide concentration did not cause any further changes in spectra. This result indicates that cyanide gives a mono-addition to the vinyl group (Scheme 2).

#### DFT results

**Figure 6:**
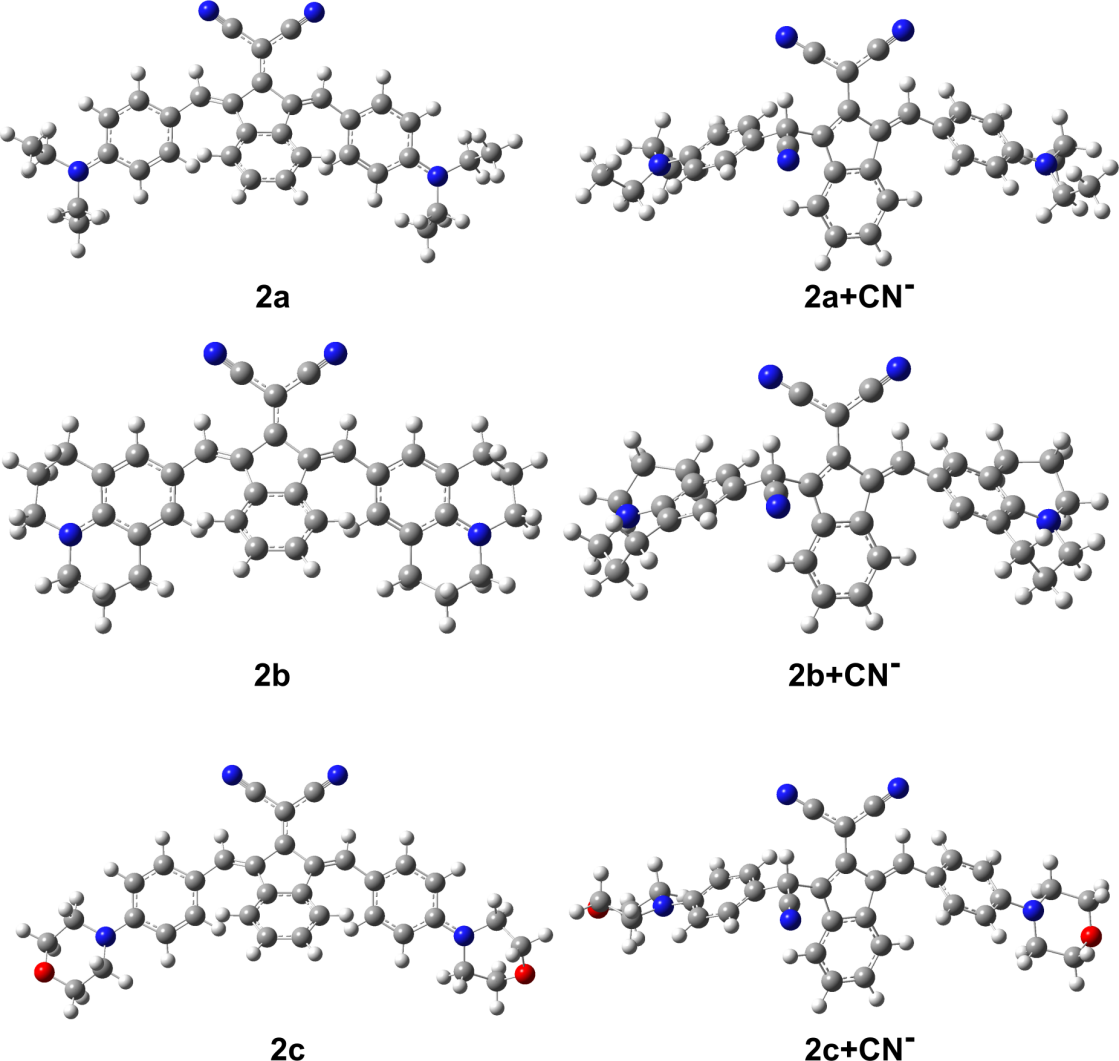
Optimized geometries of **2a**–**c** and **2a**–**c+CN^−^** obtained at the B3LYP/6-31+G(d,p) level.

To further confirm the proposed interaction mechanism involved between **2a**–**c** with CN^−^, DFT calculations were performed at B3LYP/6-31+G(d,p) level of theory. The optimized geometries **2a**–**c** and **2a**–**c+CN^−^** were represented in Figure 6.

The optimized geometries of the study’s molecules showed that π-conjugted linkers between the cyano fragments and indanone group are almost planar, with the dihedral angles with the atom-numbering in Supporting Information File 1, Figure S16, (C16–C14–C12–C13 and C15–C14–C12–C11): (0.57, −0.57) for **2a**; (0.79, −0.79) for **2b**; (0.07, −0.20) for **2c**. In addition, the dihedrals between indanone and phenyl rings (C13–C21–C23–C24 and C11–C19–C33–C34) are (157.30, −157.30); (159.29, −159.29); 154.46, −156.48) for **2a**–**c**, respectively, which is an indicator of large degrees of conjugation and good ICT. When CN^−^ interacts with the **2a**–**c** via the proposed addition mechanism (Scheme 2), the planarity between the cyano fragments and indanone group is partially reduced with the dihedrals (−26.19, −28.29) for **2a+CN**^−^, (−26.89, −28.94) for **2b+CN**^−^, (−24.77, −26.23) for **2c+CN**^−^. Furthermore, the planarity between the indanone and phenyl rings is disrupted where CN^−^ is attached, while remaining more planar on the other side, with the dihedral values (−52.13, 144.39); (−51.21, 144.32); (−51.84, 142.39) for **2a+CN**^−^, **2b+CN**^−^ and **2c+CN**^−^, respectively. As a result, conjugation is disrupted on the side where planarity is disrupted and a decrease in ICT occurs, which causes shifts in the absorption spectra.

To get further information about the electronic structures of **2a**–**c** and **2a**–**c+CN**^−^ adducts, TD-DFT calculations were performed with B3LYP/6-31+G(d,p) in DMSO. The calculated absorption maxima (λ_abs_), oscillator strengths (*f*) and corresponding transitions are given in Table 3.

**Table 3:** The absorption maxima (λ_abs_), oscillator strength (*f*) and transitions for **2a**–**c** and **2a**–**c+CN**^−^.

λ_abs_ (nm)	*f*	Transitions^a^		λ_abs_ (nm)	*f*	Transitions^a^

**2a**				**2a+CN** **^−^**		

607	0.8465	HOMO−1→LUMO		436	0.7139	HOMO−1→LUMO
422	0.7386	HOMO→LUMO+1				

**2b**				**2b+CN** **^−^**		

617	0.8785	HOMO−1→LUMO		461	0.5077	HOMO−1→LUMO
427	0.7331	HOMO→LUMO+1				

**2c**				**2c+CN** **^−^**		

572	0.5901	HOMO→LUMO		403	0.6076	HOMO−2→LUMO
412	0.7351	HOMO→LUMO+1				

^a^HOMO: highest occupied molecular orbital, LUMO: lowest unoccupied molecular orbital.

**Figure 7:**
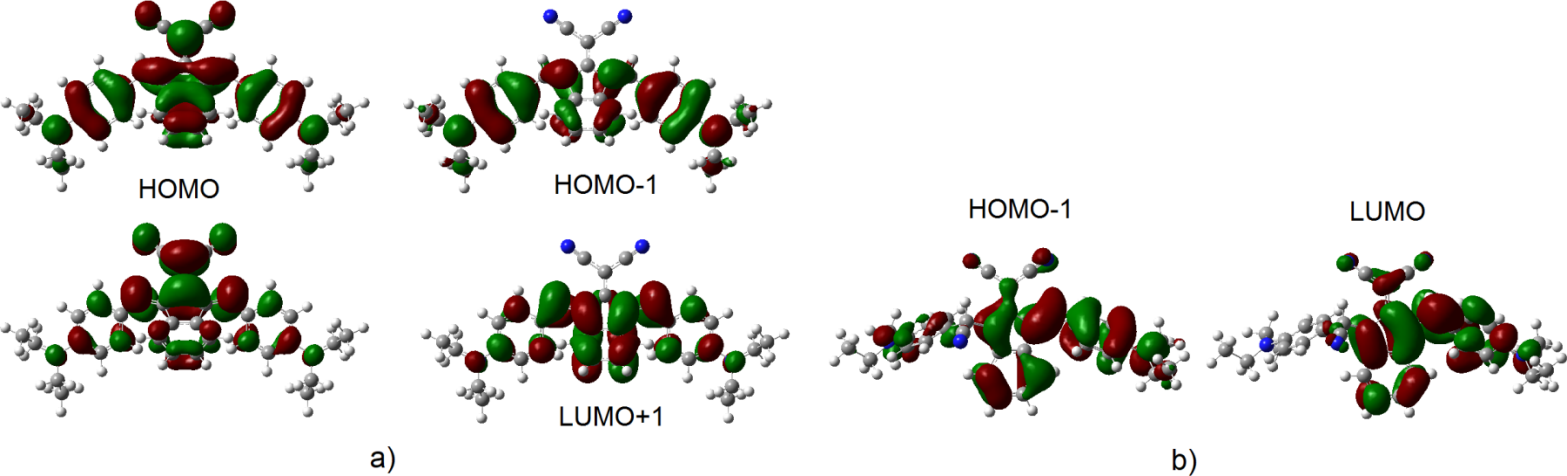
Frontier molecular orbitals of a) **2a,** b) **2a+CN**^−^.

As given in Table 2, the obtained absorption wavelengths at 422 nm (427 nm) and 607 nm (617 nm) for **2a** (**2b**) had significant contributions (99%) from HOMO−1→LUMO and HOMO→LUMO+1, respectively. For **2c**, the major contributions come from HOMO→LUMO+1 (96%) and HOMO→LUMO (96%) for the transitions at 412 nm and 572 nm, respectively. The electron distribution is located at the donor groups and slightly at the dicyanovinylene unit in the HOMO. The distribution in the HOMO−1 mainly is over the donor groups (Figure 7 and Figure S17, Supporting Information File 1). The LUMO is delocalized over the dicyanovinylene unit and slightly over the indanone group, while the LUMO+1 is over the indanone group. From this, it is predicted that there is an ICT from donor groups to acceptor groups, resulting in absorption maxima in the spectrum.

In case of **2a**–**c+CN**^−^ adducts formed after the interaction with CN^−^ anion with the studied molecules, a single peak appears at 436 nm, 461 nm and 403 nm, respectively, and the peak at the longer wavelength seen before interaction with CN^−^ disappeared. The peaks for **2a**–**b+CN**^−^ arise from the HOMO−1→LUMO transitions, while for **2c+CN**^−^ are contributed from the HOMO−2→LUMO transitions, with the contributions ≈94–99%.

Considering the molecular orbitals reveal that there are no contributions from the side where the planarity is disrupted after the addition of the CN^−^ anion and the electron distributions shift towards the other side where conjugation exists (Figure 7 and Figure S17, Supporting Information File 1).

#### NLO properties

The EFISH method and DFT calculation were used to examine the NLO responses of **2a**–**c**. Measurements were made using the EFISH method in a CHCl_3_ solution at a non-resonant incident wavelength of 1907 nm. Experimental details for the EFISH measurements and results are presented in Table 4 [32]. The excited states were more polarized than the ground states, as shown by the positive observed μβ values. Moreover, both the ground and excited states were polarized in the same direction for all studied compounds. The standard reference is Disperse Red 1 (μβ = 450 × 10^−48^ esu) [33]. Comparing the µβ values of Disperse Red 1 with the measured values of **2a**–**c** shows that the molecules exhibited a higher NLO response than Disperse Red 1, except for **2c**.

**Table 4:** Experimental and calculated NLO properties and energy gap (Δ*E*) values for **2a–c**.

Dyes	µβ^a^(×10^−48^) (esu)	Δ*E*^b^ (eV)	µ^b^ (D)	α^b^(×10^−24^) (esu)	β^b^(×10^−30^) (esu)

**2a**	1170	2.36	15	117	404
**2b**	1740	2.32	15	116	389
**2c**	300	2.42	12	113	362

^a^EFISH: μβ (2ω) at 1907 nm in CHCl_3_, molecular concentrations used for the measurements were in the range of 10^−3^ to 10^−2^ M. μβ ± 10%. ^b^DFT results at the B3LYP/6-31+G(d,p) level of theory in CHCl_3_.; esu: electrostatic unit.

The dipole moment (μ), polarizability (α), first-order hyperpolarizability (β), and its components, which were calculated at the B3LYP/6-31+G(d,p) level of theory in CHCl_3_, are also included in Table 4 and Table S1, Supporting Information File 1. According to the calculations, there is more charge delocalization in the xxy direction because the βxxy component is larger (Table S1, Supporting Information File 1). A significant first-order hyperpolarizability value (β) is indicative of a high NLO response in a typical organic NLO chromophore. Apart from the existence of donor (D) and acceptor (A) groups linked by a π-conjugation path, the NLO response is also influenced by the strength of the D and A groups in the structure. For high NLO responses, a small energy gap between the HOMO and the LUMO (Δ*E*), resulting from the presence of the strong D/A groups, is an important indicator. Based on the EFISH results, the calculations show that **2a** and **2b** have a small energy gap and a high β value because they have a stronger electron-donor group than **2c**. Additionally, **2a** and **2b** have higher polarizability values and dipole moments (μ) than **2c**.

#### TGA analysis

Thermogravimetric analyses (TGA) were performed to determine the thermal stability of the dyes. The TGA method allows the determination of thermal and gravimetric changes in the material following temperature increases. Dyes, especially those with potential for use in optical systems, must be stable up to certain temperatures depending on the systems [34–36].

**Figure 8:**
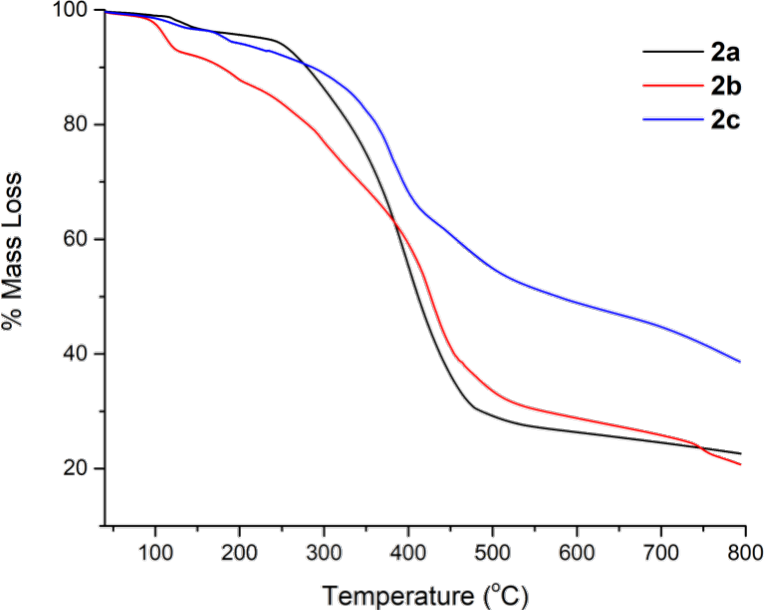
TGA curves of dyes.

The percentage mass loss versus temperature graph is shown in Figure 8. The decomposition temperatures (*T*_d_) of the dyes are given in Table 5. Results show that the dyes are in the range of 254–339 °C.

**Table 5:** *T*_d_ values of the dyes.

Dyes	*T*_d_ (°C) (%85)

**2a**	263
**2b**	254
**2c**	339

The mass loss between 0 and 150 °C indicates the presence of water or an organic solvent in the structure of compound **2b**. The *T*_d_ values of the dyes are generally resistant to moderate to high temperatures. This result supports their potential use as optical dyes.

## Conclusion

In summary, we have determined optical and chemosensor properties of symmetrical D−A dyes **2a**, **2b**, and **2c**. We found that strong electron-donating properties and increased planar structure of julolidine (**2b**) as a donor induced a significant bathochromic shift to the NIR region and the greatest extinction coefficient Also, dyes exhibit positive solvatochromism, consistent with their ICT characteristics. Moreover, synthesized symmetric dyes containing D–π–A–π–D systems were analyzed using experimental and computational methods for second-order nonlinear optical properties. NLO measurements conducted using the EFISH method, where μβ values were found between 300 × 10^−48^ esu and 1740 × 10^−48^ esu and the highest value observed was with **2b**. Dyes also showed chemosensor properties for the selective detection of cyanide with colorimetric and optical responses in both organic and aqueous media. The interaction mechanism is determined as mono-cyanide addition to the vinyl group via Michael reaction. These results show that symmetrical indanone-based D–π–A–π–D dyes can be used in future optoelectronic devices and environmental monitoring.

## Supporting Information

File 1 Synthesis of compounds, copies of NMR, HRMS and UV–vis spectra and DFT results.

## Data Availability

All data that supports the findings of this study is available in the published article and/or the supporting information of this article.
