# Supplementary material for: Symmetrical D–π–A–π–D indanone dyes: a new design for nonlinear optics and cyanide detection
Source: Beilstein J Org Chem. 2026 Jan 14;22:131–42. doi: 10.3762/bjoc.22.6 (PMC12816989; doi:10.3762/bjoc.22.6)
Supplement: File 1 — Synthesis of compounds, copies of NMR, HRMS and UV–vis spectra and DFT results. [file Beilstein_J_Org_Chem-22-131-s001.pdf]

BEILSTEIN JOURNAL OF ORGANIC CHEMISTRY

Supporting Information

for

Symmetrical D–π–A–π–D indanone dyes: a new design for nonlinear optics and cyanide detection

Ergin Keleş, Alberto Barsella, Nurgül Seferoğlu, Zeynel Seferoğlu and Burcu Aydıner

Beilstein J. Org. Chem. **2026,** *22,* 131–142. doi:10.3762/bjoc.22.6

Synthesis of compounds, copies of NMR, HRMS and UV–vis spectra and DFT results

License and Terms: This is a supporting information file under the terms of the Creative Commons Attribution License (https://creativecommons.org/licenses/by/4.0). Please note that the reuse, redistribution and reproduction in particular requires that the author(s) and source are credited and that individual graphics may be subject to special legal provisions.
The license is subject to the *Beilstein Journal of Organic Chemistry* terms and conditions: (https://www.beilstein-journals.org/bjoc/terms)

Table of Contents

Materials and methods S1

Synthesis S2

Copy of NMR and HRMS spectra S6

UV–vis Spectra S11

DFT Results S16

References S18

Materials and methods

All commercially available chemicals were reagent grade and used without further purification. Thin-layer chromatography (TLC) was used for monitoring the reactions using precoated silica gel 60 F254 plates. NMR spectra were measured on Bruker Avance NEO 500 (^{1}H: 500 MHz, ^{13}C:126 MHz) spectrometer at 20 °C (293 K). Chemical shifts (δ) are given in parts per million (ppm) using the residue solvent peaks as a reference relative to TMS. Coupling constants (*J*) are given in hertz (Hz). Signals are abbreviated as follows: singlet. s; doublet. d; doublet-doublet. dd; triplet. t; quintet. quint. High-resolution mass spectra (HRMS) were recorded at Bilkent University National Nanotechnology Research Center using Agilent Technologies 6530 Accurate-Mass Q-TOF LC/MS. The microwave syntheses were carried out in a Milestone Start. The melting points were measured using Electrothermal IA9200 apparatus. Horiba Duetta spectrophotometer was used for absorption.

Photophysical properties

For photophysical studies, 1 mM stock solutions of the synthesized dyes were prepared in solvents of different polarity (DMSO, Acetone, Chloroform, and THF).

Anion interaction studies

Stock solutions of the dyes (1 mM) were prepared in DMSO. Stock solutions of anions (F^-, Cl^-, Br^-, I^-, AcO^-, CN^-, ClO_4^-, $H_2PO_4^-$, HSO_4^-, and NO_3^-, as tetrabutylammonium salts) were prepared in DMSO at a concentration of 10 mM.

NMR titration studies

^{1}H NMR titration experiments were performed to investigate the interaction of the dyes with anions. Stock solutions of CN^- (1 M, as tetrabutylammonium salt) and dye

solutions (10 mM) were prepared in DMSO-d_6. Aliquots of the two solutions were directly mixed in NMR tubes for titration.

DFT calculation methods

The ground-state geometries were obtained by performing density functional theory (DFT) at B3LYP/6-31+G(d,p) level [1–3]. The optimized structures were characterized as true minima by vibrational frequency calculations. The electronic absorption spectra were obtained within the time-dependent DFT (TD-DFT) methods at the same level in DMSO using the PCM model [4,5]. All the DFT calculations were performed using the Gaussian 09 software [6].

Synthesis

*Synthesis of 2-(1,3-dihydro-2H-inden-2-ylidene)malononitrile (**1**):* The solution of 20 mmol (2.64 g) indan-2-one, 22 mmol (1.45 g) malononitrile, 4 mmol (0.308 g) ammonium acetate, and 9.4 mmol (538 µL) acetic acid mixture in 30 mL DCM were refluxed for half an hour. The solvent was evaporated. The product was mixed with water and washed with methanol. The obtained solid was crystalized with ethanol [7]. Green light crystal, yield 84% (3 g) m.p.: 192–194 ºC.

General synthesis of compounds 2a-c:

Conventional method (CM):

1 mmol of compound 1 (0.180 g) and 3.6 mmol of the appropriate aldehyde derivative were dissolved in 5 mL of acetic anhydride and refluxed under a nitrogen atmosphere under stirring. After completion, the reaction mixture was cooled to room temperature, and the solvent was removed under reduced pressure, and the crude product was purified by an appropriate purification method.

Microwave Irradiation (MWI) method:

0.5 mmol of compound 1 (0.09 g) and 1.8 mmol of the corresponding aldehyde derivative were dissolved in 5 mL of acetic anhydride. The reaction was carried out using microwave irradiation (MWI, 500 W) at 135 °C for 4.5 minutes. After completion, the reaction mixture was cooled to room temperature, and the solvent was removed under reduced pressure, and the crude product was purified by an appropriate purification method.

*2-(1,3-Bis((E)-4-(diethylamino)benzylidene)-1,3-dihydro-2H-inden-2-ylidene)malononitrile (**2a**):*

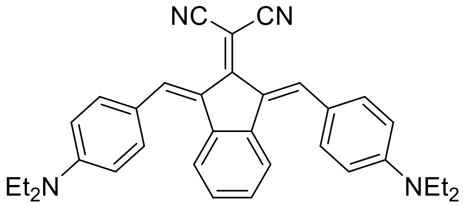

Reaction time: 45 min. The compound was crystallized from petroleum ether, yielding a dark purple solid. m. p.: 106-107 °C. Yield according to CM: 75%; method MWI: 62%. ^{1}H NMR (500 MHz, $CDCl_3$) δ 8.30 (s, 1H), 7.90 (dd, *J* = 5.9, 3.3 Hz, 1H), 7.64 (d, *J* = 8.8 Hz, 2H), 7.06 (dd, *J* = 5.9, 3.1 Hz, 1H), 6.68 (d, *J* = 9.0 Hz, 2H), 3.47 (q, *J* = 7.1 Hz, 4H), 1.26 (t, *J* = 7.1 Hz, 6H). ^{13}C APT NMR (126 MHz, $CDCl_3$) δ 169.6, 149.3, 138.0, 136.5, 132.4, 131.3, 127.3, 122.5, 121.4, 116.5, 111.5, 110.8, 44.5, 12.7. HRMS (*m/z*), $(M+H)^+$: $C_{34}H_{34}N_4$, calculated: 499.28617; found: 499.28618.

*2-((1E,3E)-1,3-Bis((2,3,6,7-tetrahydro-1H,5H-pyrido[3,2,1-ij]quinolin-9-yl)methylene)-1,3-dihydro-2H-inden-2-ylidene)malononitrile (**2b**):*

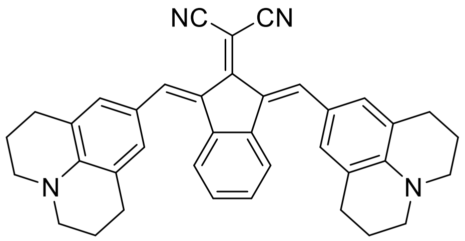

Reaction time: 30 min. The compound was purified by column chromatography using dichloromethane as the eluent. A dark green solid was obtained. m. p.: 208-210 °C (decomp). Yield: CM: 46%; method MWI: 45%. ^{1}H NMR (500 MHz, $CDCl_3$) δ 8.25 (s, 1H), 7.95 (dd, *J* = 5.9, 3.2 Hz, 1H), 7.27 (s, 2H), 7.03 (dd, *J* = 5.9, 3.2 Hz, 1H), 3.32 (t, *J* = 6.4 Hz, 4H), 2.76 (t, *J* = 6.4 Hz, 4H), 2.04 (quint, *J* = 6.3 Hz, 4H). ^{13}C NMR (126 MHz, $CDCl_3$) δ 169.0, 145.0, 138.2, 137.2, 130.8, 129.9, 126.9, 122.1, 121.4, 120.6, 117.1, 50.1, 27.5, 21.5. HRMS (*m/z*), $(M+H)^+$: $C_{38}H_{35}N_4$, calculated: 547.28617; found: 547.28648.

*2-(1,3-Bis((E)-4-morpholinobenzylidene)-1,3-dihydro-2H-inden-2-ylidene)malononitrile (**2c**)*

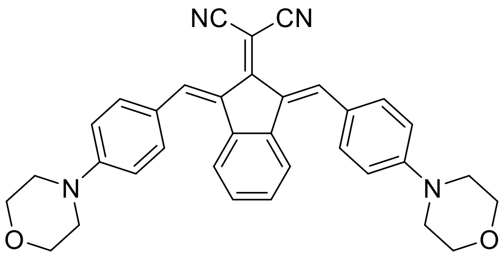

Reaction time: 2 h. The compound was purified by column chromatography using a dichloromethane:petroleum ether (1:1, v/v) solvent system. A black solid was obtained. m. p.: 185-188 °C. Yield: CM: 22% (0.115 g); method MWI: 25% (0.065 g). ^{1}H NMR (500 MHz, $CDCl_3$) δ 8.25 (s, 1H), 7.95 (dd, *J*=5.9, 3.2 Hz, 1H), 7.27 (s, 2H), 7.03 (dd,

J=5.9, 3.2 Hz, 1H), 3.32 (t, *J*=6.4 Hz, 4H), 2.76 (t, *J*=6.4 Hz, 4H), 2.04 (quint, *J*=6.3 Hz, 4H). ^{13}C APT NMR (126 MHz, $CDCl_3$) δ 169.0, 145.0, 138.2, 137.2, 130.8, 129.9, 126.9, 122.1, 121.4, 120.6, 117.1, 50.1, 27.5, 21.5. HRMS (*m/z*), $(M+H)^+$: $C_{38}H_{35}N_4$, calculated: 527.24470; found: 527.24453.

Copy of NMR and HRMS spectra

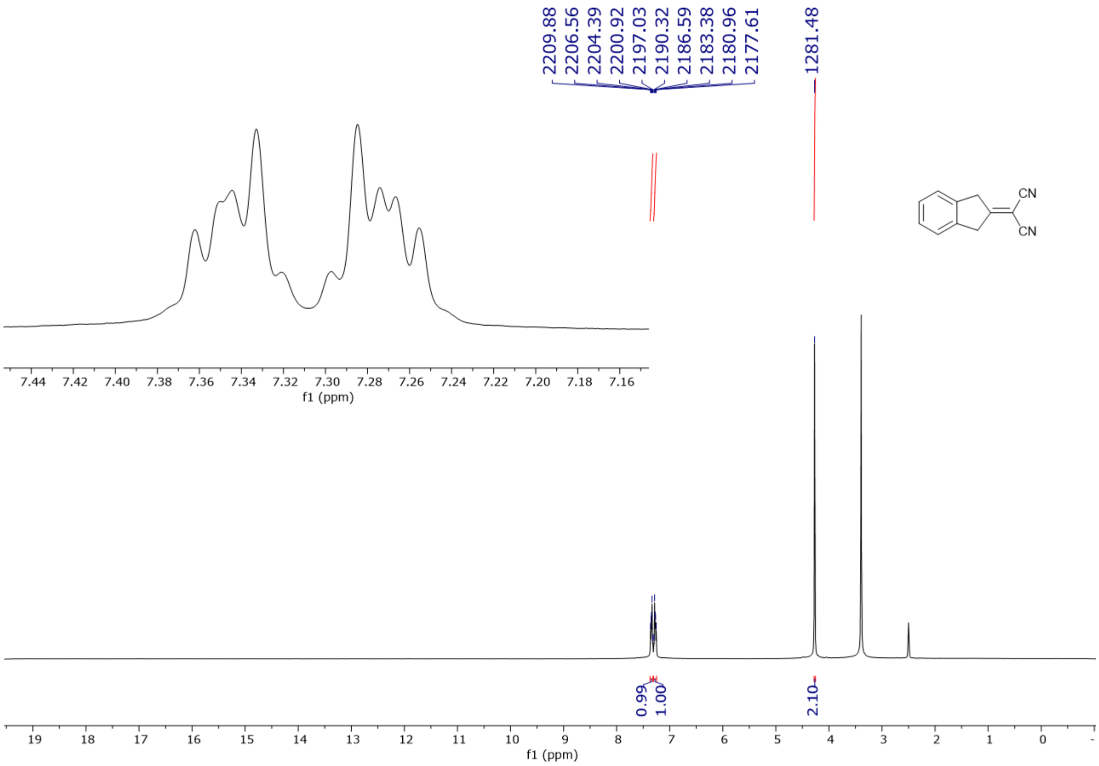

Figure S1. The ^{1}H NMR spectra of compound **1** in DMSO-d_6.

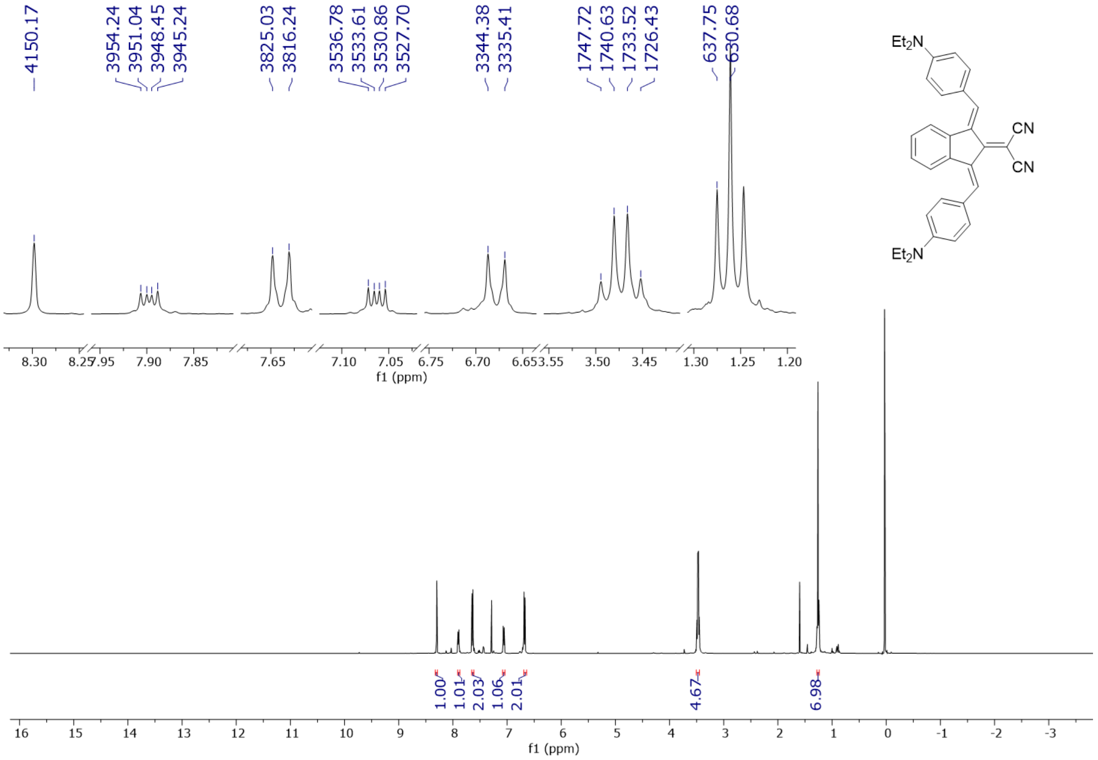

Figure S2. The ^{1}H NMR spectra of compound **2a** in $CDCl_3$.

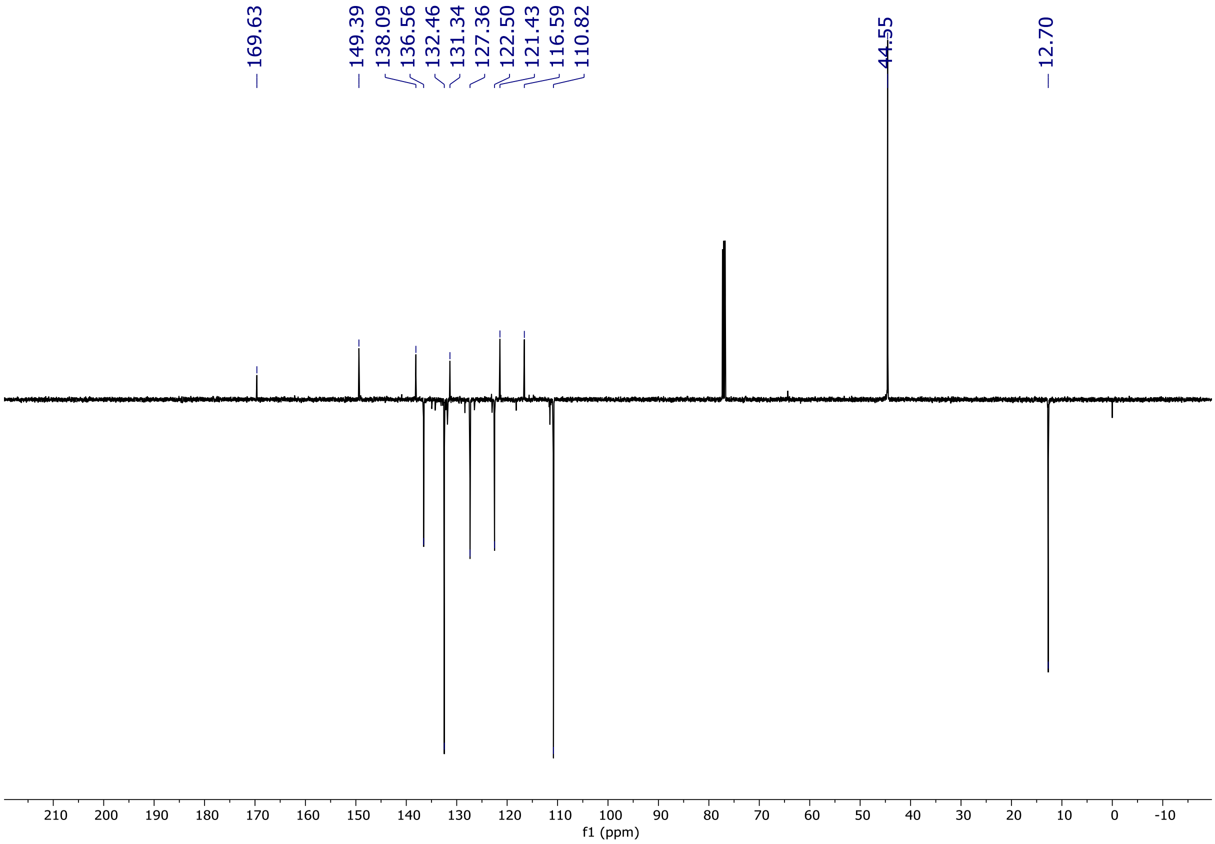

Figure S3. The ^{13}C APT NMR spectra of compound **2a** in $CDCl_3$.

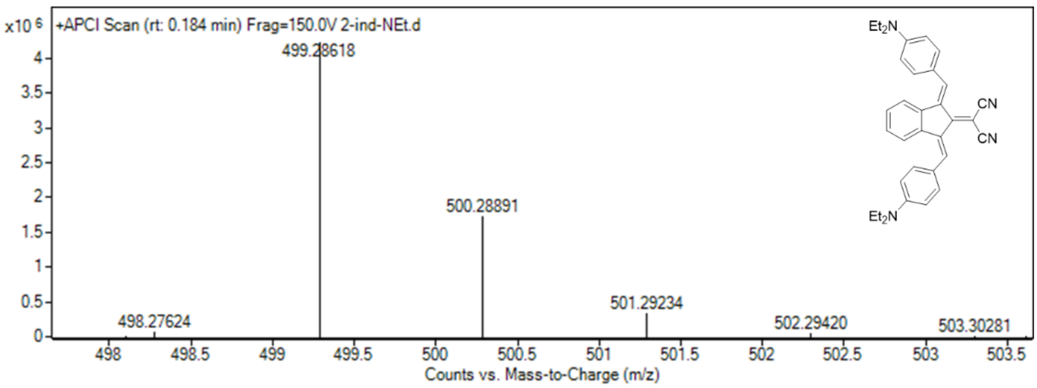

Figure S4. The mass spectra of compound **2a**.

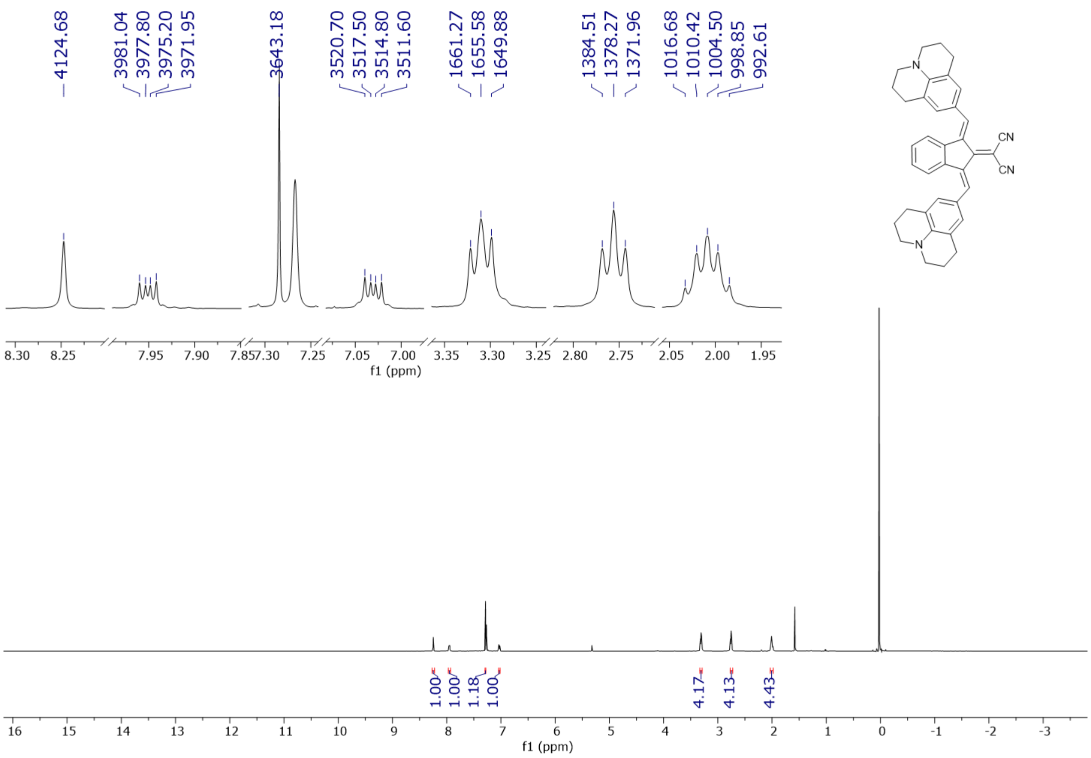

Figure S5. The ^{1}H NMR spectra of compound **2b** in $CDCl_3$.

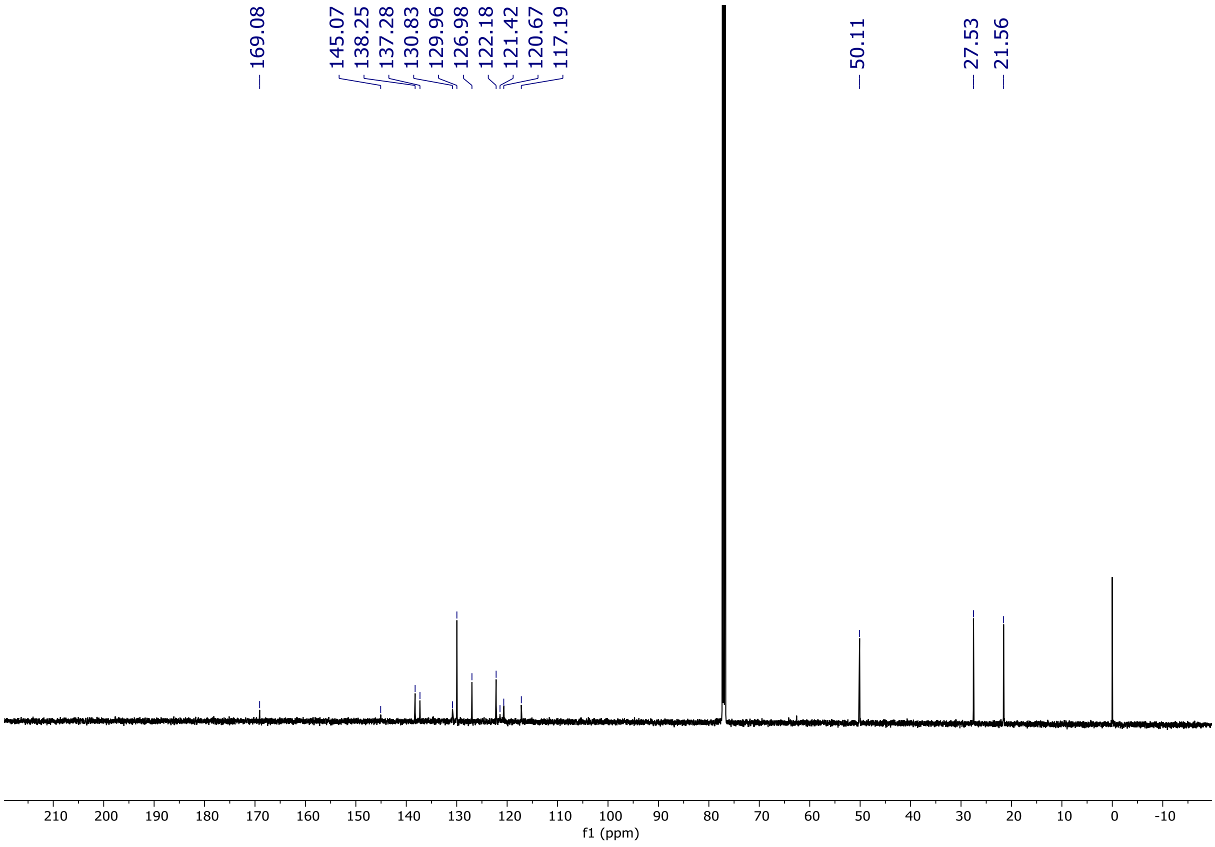

Figure S6. The ^{13}C NMR spectra of compound **2b** in $CDCl_3$.

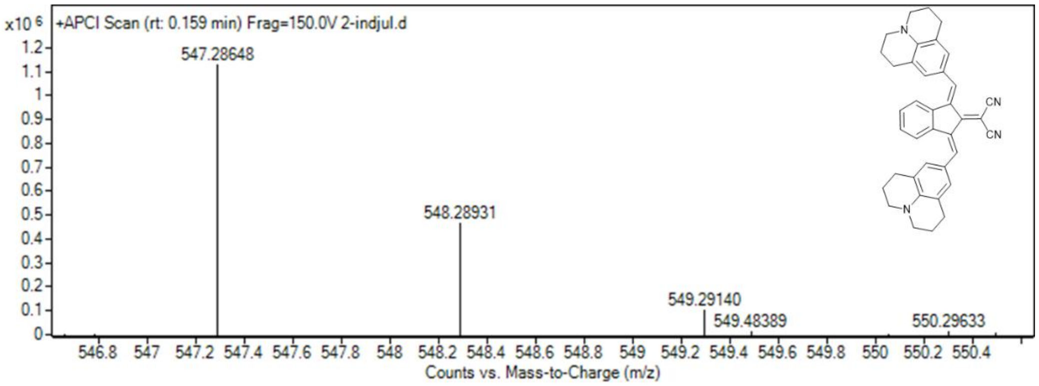

Figure S7. The mass spectra of compound **2b**.

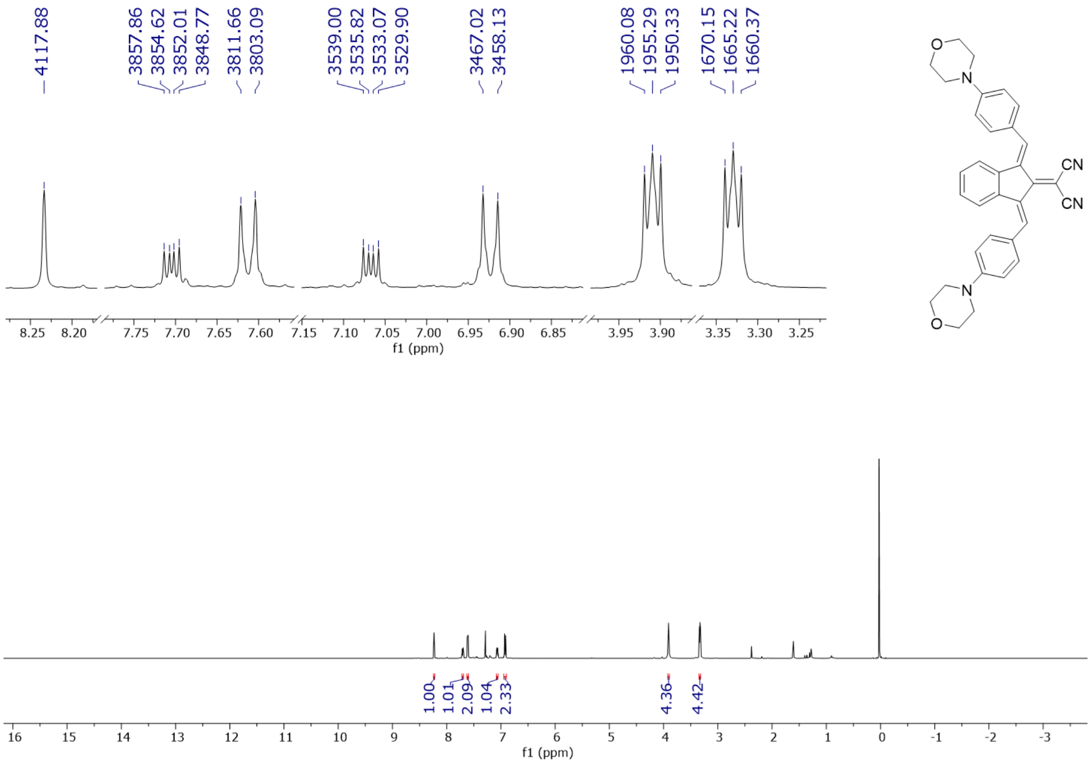

Figure S8. The ^{1}H NMR spectra of compound **2c** in $CDCl_3$.

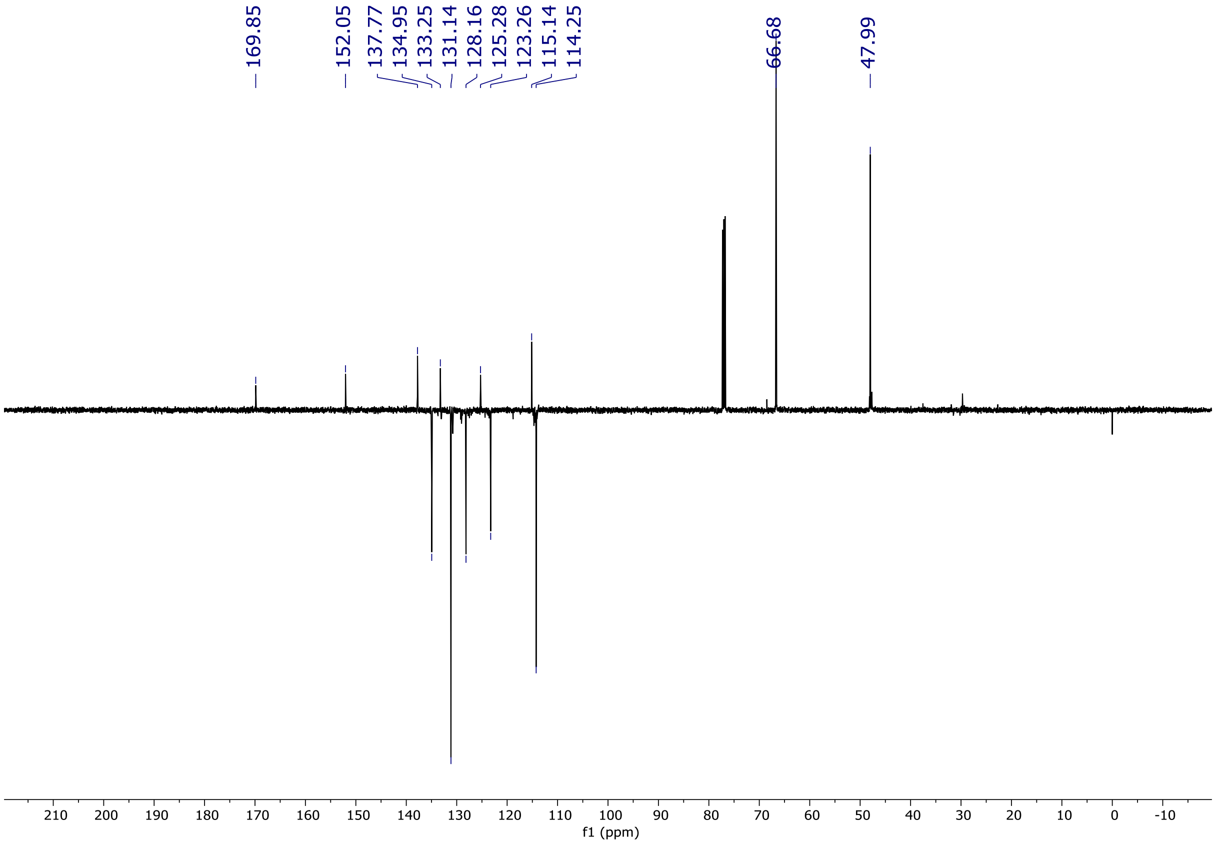

Figure S9. The ^{13}C APT NMR spectra of compound **2c** in $CDCl_3$.

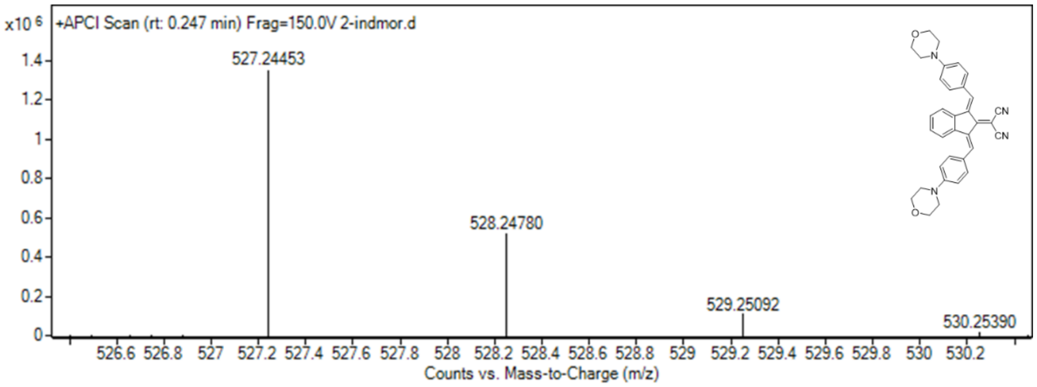

Figure S10. The mass spectra of compound **2c**.

UV–vis Spectra

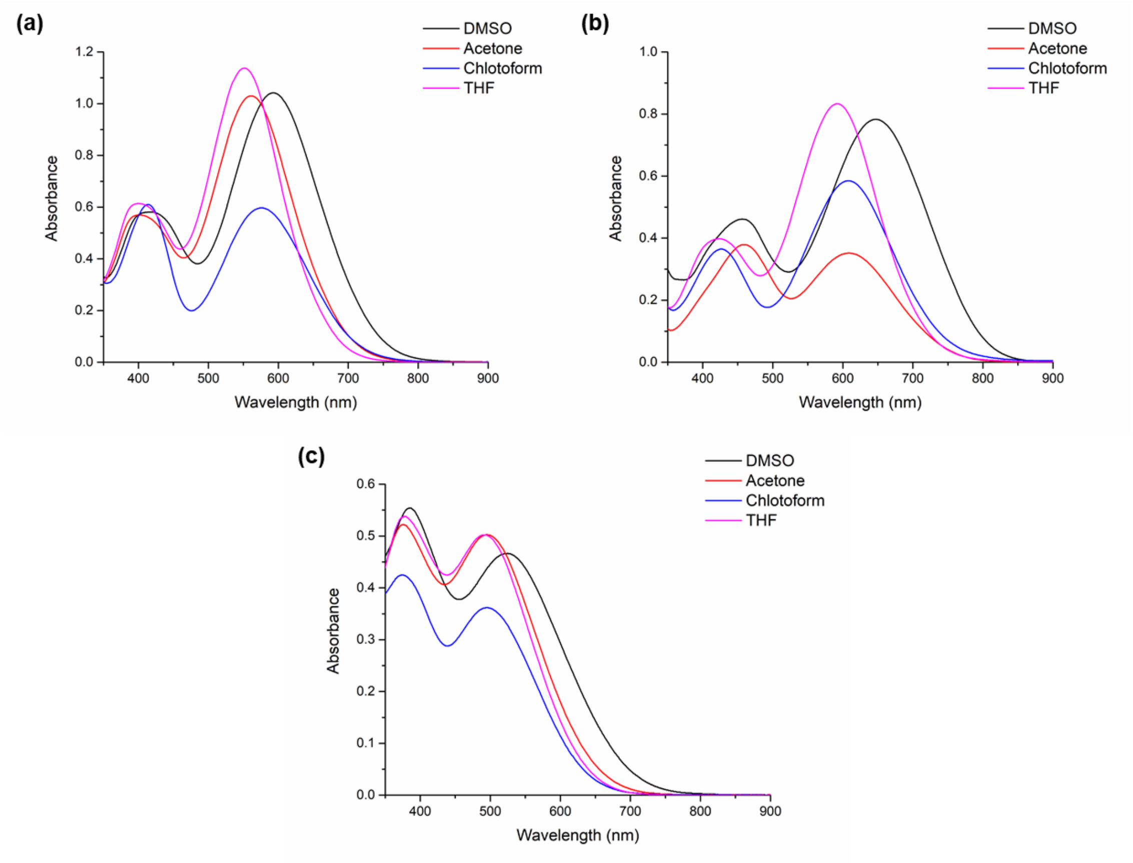

Figure S11. Absorption spectra of compounds **2a** (a), **2b** (b), and **2c** (c) recorded in different solvents at a concentration of 10 μM.

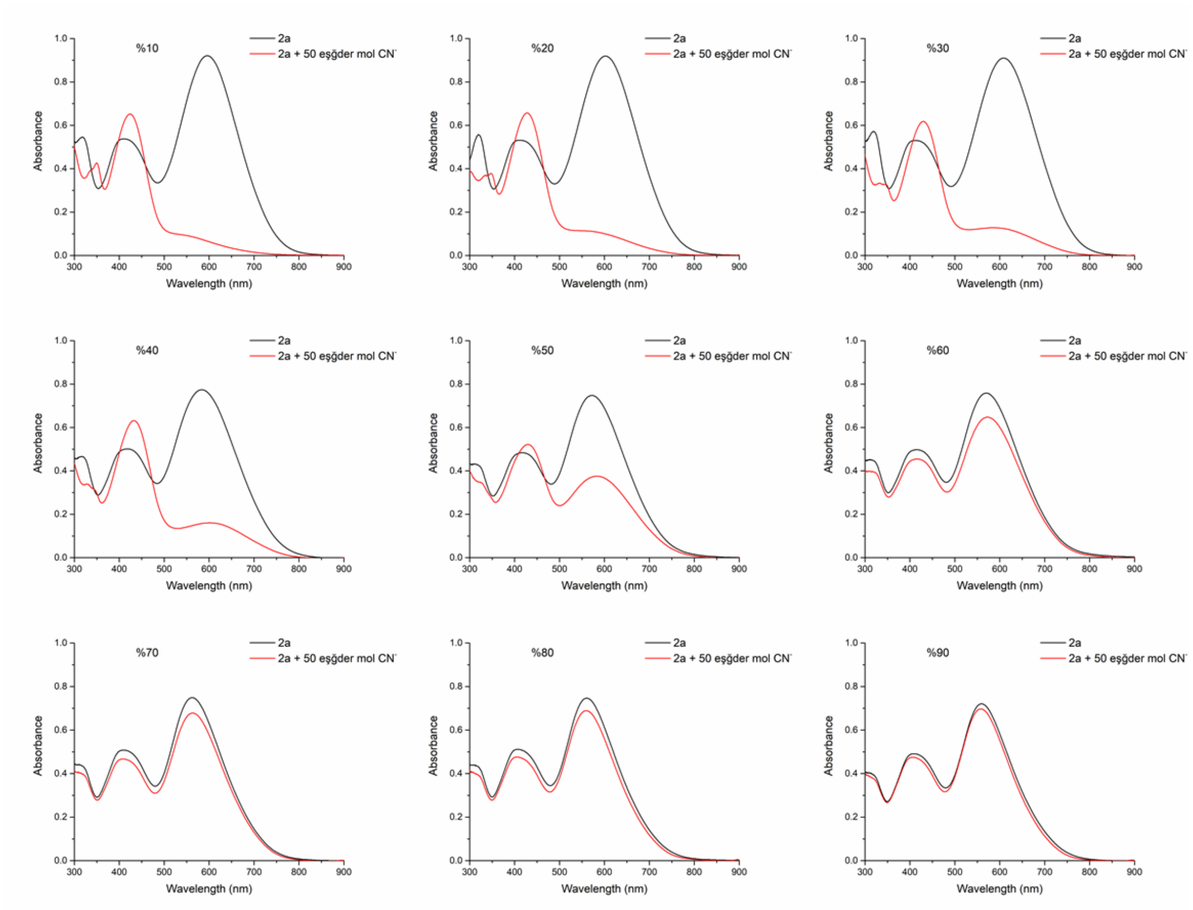

Figure S12. Absorption spectra of compound **2a** (*c* = 30 μM) in DMSO:H_2O (v/v) mixtures with increasing water fractions (10–90%) upon the addition of 50 equivalents of TBACN.

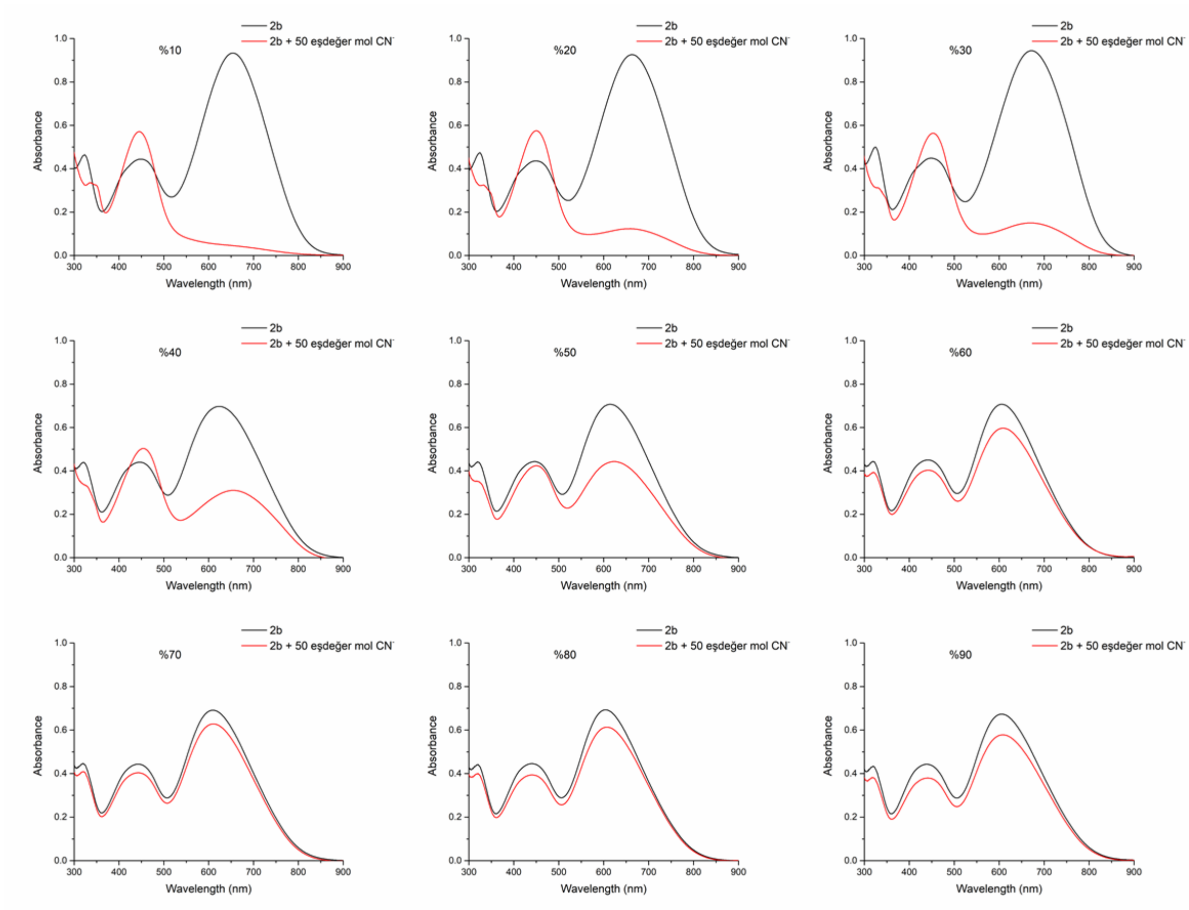

Figure S13. Absorption spectra of compound **2b** (*c* = 30 µM) in DMSO:H_2O (v/v) mixtures with increasing water fractions (10–90%) upon the addition of 50 equivalents of TBACN.

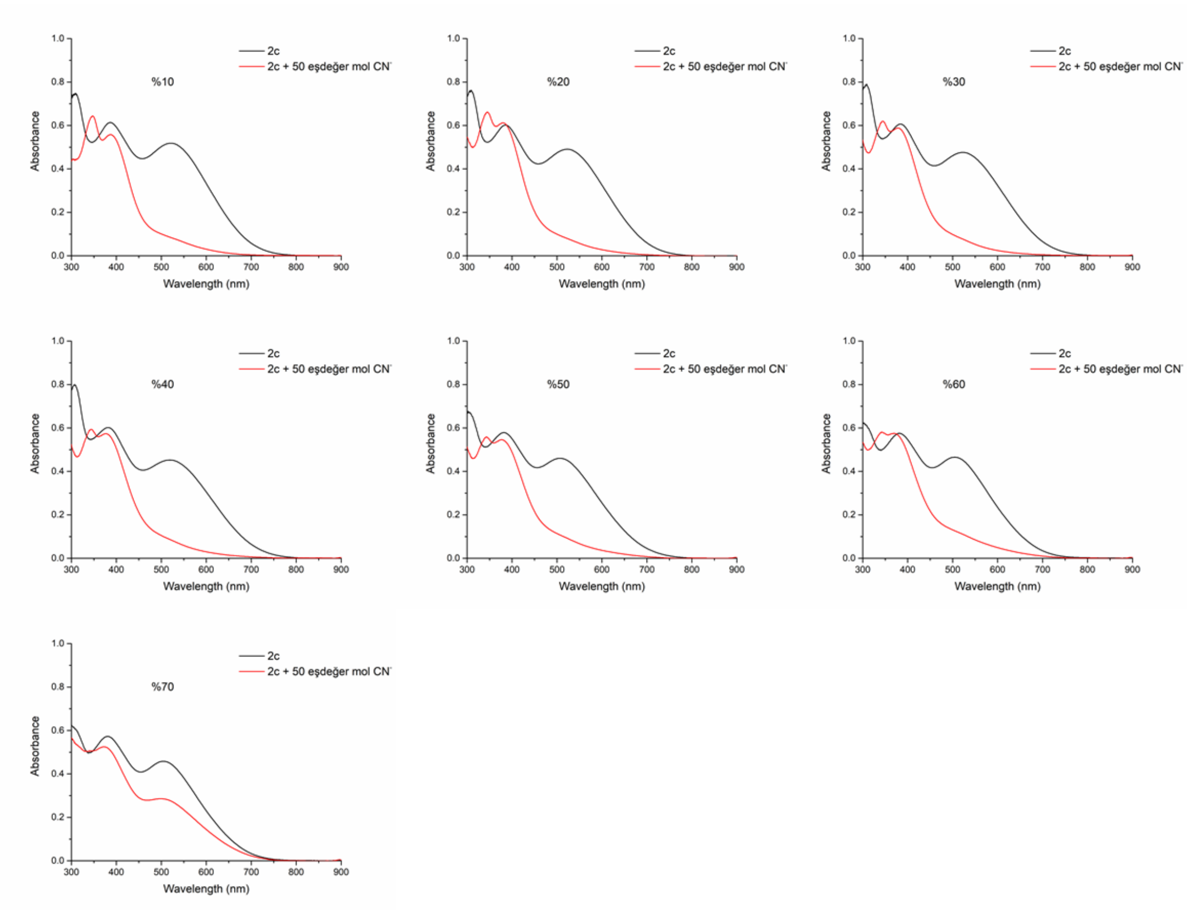

Figure S14. Absorption spectra of compound **2c** (*c* = 30 μM) in DMSO:H_2O (v/v) mixtures with increasing water fractions (10–70%) upon the addition of 50 equivalents of TBACN.

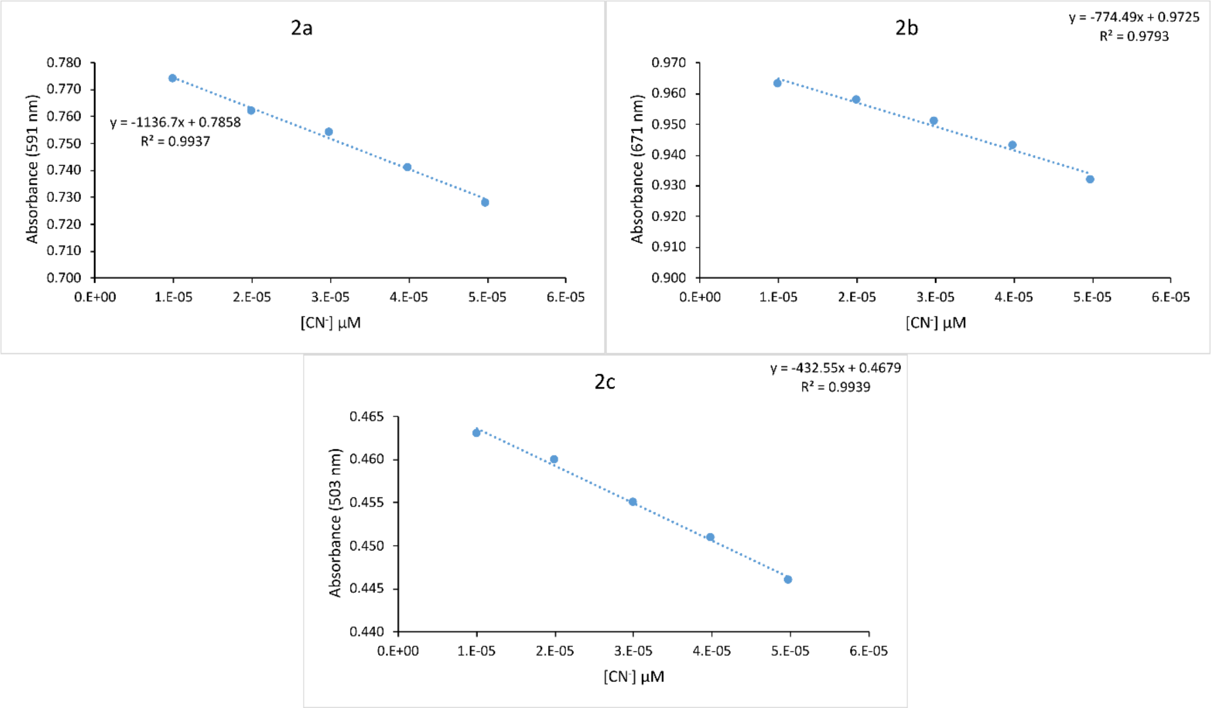

Figure S15. The absorption calibration curve of CN^- in DMSO for compounds.

DFT Results

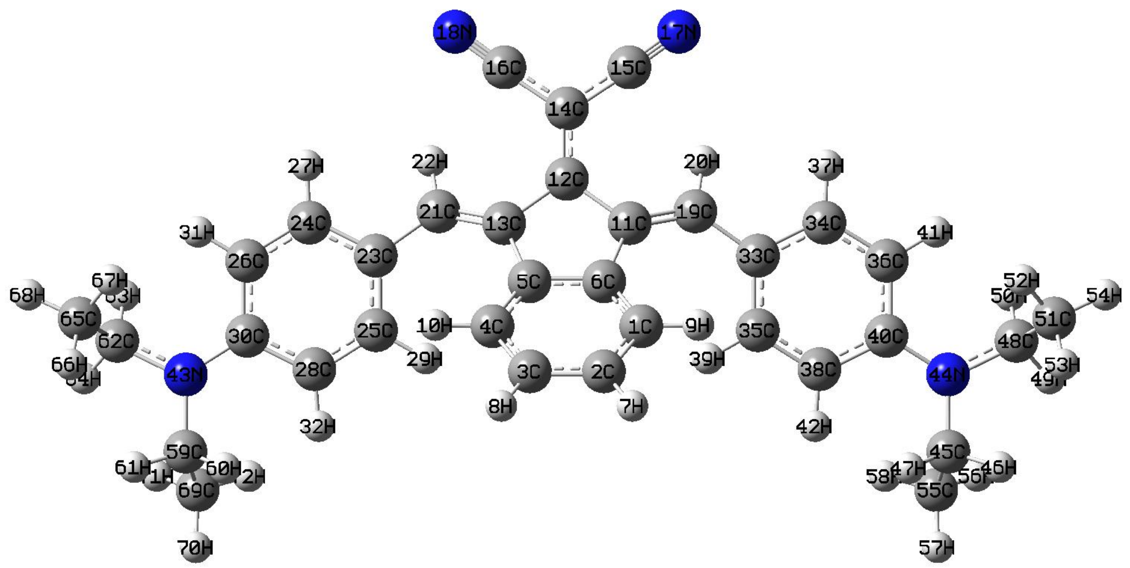

Figure S16. Optimized geometry of **2a** with the atom-numbering.

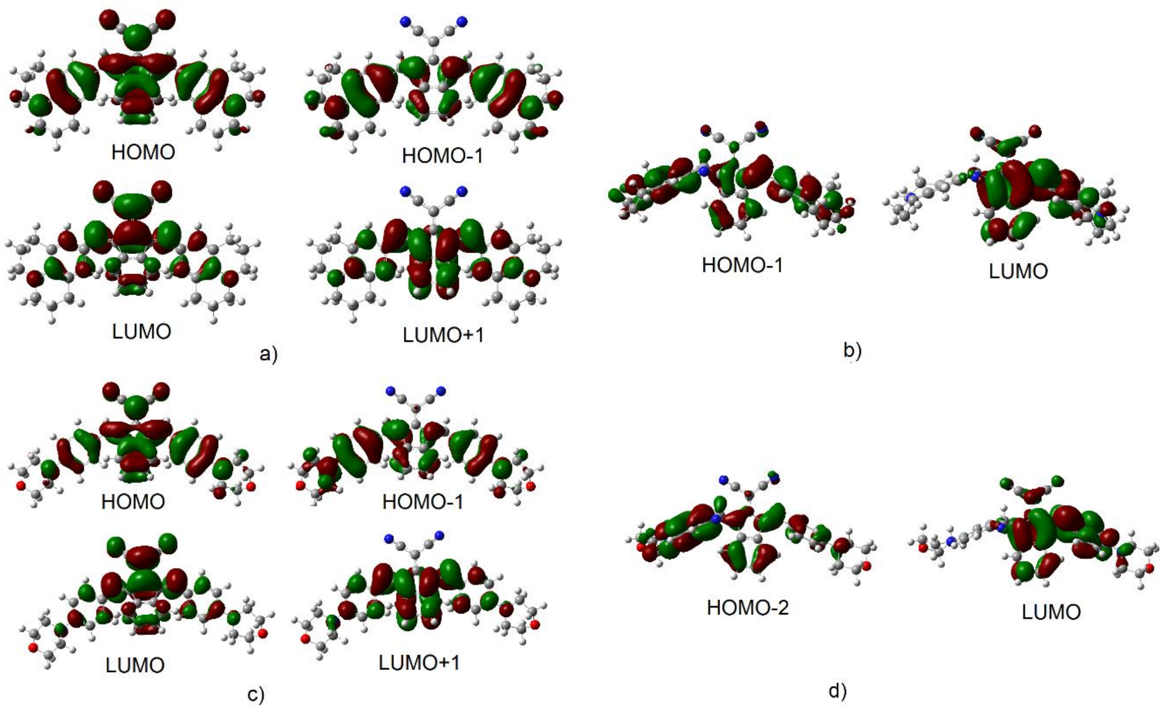

Figure S17. Frontier molecular orbitals of a) **2b**, b) **2b+CN**$^-$, c) **2c**, d) **2c+CN**$^-$.

Table S1. The calculated dipole moment (μ, in D), first-order hyperpolizability (β, in esu) and polarizability (α, in esu) with their components (in a.u) for 2a-c using B3LYP/6-31+g(d,p) in $CHCl_3$

	**2a**	**2b**	**2c**
μ_x	-0.00012	-0.0002629	0.5838721
μ_y	-5.9732232	-5.7130393	-4.6944411
μ_z	0.1654552	-0.8378511	0.134385
**μ (D)**	**15**	**15**	**12**
α_{xx}	1292.917099	1340.198526	1225.280946
α_{xy}	0.0061971	-0.0069701	-24.2748585
α_{yy}	694.2399054	688.3537101	681.4925729
α_{xz}	0.0058909	-0.0047136	-6.4687292
α_{yz}	-41.1417606	-9.9557798	-42.3103384
α_{zz}	377.472268	316.4788132	373.1236449
**α(x10^{-24})(esu)**	**117**	**116**	**113**
β_{xxx}	1.9913006	3.1765027	-5145.99916
β_{xxy}	41907.61238	40141.39148	36419.20373
β_{xyy}	46.390366	25.1402657	-1994.94303
β_{yyy}	4522.394098	3115.74797	4665.966809
β_{xxz}	4031.250305	10628.61191	4655.711306
β_{xyz}	-1.9021083	-1.0039282	-299.833506
β_{yyz}	1006.556371	1267.526136	1083.544672
β_{xzz}	1.7876538	-1.3210963	-337.446284
β_{yzz}	11.5510843	140.845478	-337.446284
β_{zzz}	252.5056775	168.7181681	210.5094488
**β(x10^{-30})(esu)**	**404**	**389**	**362**

References

(1) Becke, A. D. *J Chem Phys* **1993**, *98*, 5648–5652. doi:10.1063/1.464913

(2) Miehlich, B.; Savin, A.; Stoll, H.; Preuss, H. *Chem Phys Lett* **1989**, *157*, 200–206. doi:10.1016/0009-2614(89)87234-3

(3) Lee, C.; Yang, W.; Parr, R. G. *Phys Rev B* **1988**, *37*, 785. doi:10.1103/PhysRevB.37.785

(4) Cossi, M.; Barone, V. *J Chem Phys* **2001**, *115*, 4708. doi:10.1063/1.1394921

(5) Bauernschmitt, R.; Ahlrichs, R. *Treatment of Electronic Excitations within the Adiabatic Approximation of Time Dependent Density Functional Theory*; 1996; Vol. 256

(6) M.J. Frisch, G.W. Trucks, H.B. Schlegel, G.E. Scuseria, M.A. Robb, J.R. Cheeseman, G. Scalmani, V. Barone, B. Mennucci, G.A. Petersson, H. Nakatsuji, M. Caricato, X. Li, H.P. Hratchian, A.F. Izmaylov, J. Bloino, G. Zheng, J.L. Sonnenberg, M. Hada, M. Ehar, D. J. F. Gaussian 09, Revision C.01, Gaussian, Inc., Wallingford CT, 2010.

(7) Espacenet – search results https://worldwide.espacenet.com/patent/search/family/021985951/publication/GB988627A?q=pn%3DGB988627A (accessed Sep 22, 2025)
